# Turbo‐charging crop improvement: harnessing multiplex editing for polygenic trait engineering and beyond

**DOI:** 10.1111/tpj.70527

**Published:** 2025-10-15

**Authors:** Kangquan Yin, Chung‐Jui Tsai

**Affiliations:** ^1^ Center for Bioenergy Innovation Oak Ridge National Laboratory Oak Ridge Tennessee 37831 USA; ^2^ School of Forestry and Natural Resources University of Georgia Athens Georgia 30602 USA; ^3^ Department of Genetics University of Georgia Athens Georgia 30602 USA; ^4^ Department of Plant Biology University of Georgia Athens Georgia 30602 USA

**Keywords:** CRISPR‐Cas, multiplex genome editing, polygenic trait stacking, combinatorial mutagenesis, chromosomal engineering, de novo domestication, synthetic biology

## Abstract

Multiplex CRISPR editing has emerged as a transformative platform for plant genome engineering, enabling the simultaneous targeting of multiple genes, regulatory elements, or chromosomal regions. This approach is effective for dissecting gene family functions, addressing genetic redundancy, engineering polygenic traits, and accelerating trait stacking and *de novo* domestication. Its applications now extend beyond standard gene knockouts to include epigenetic and transcriptional regulation, chromosomal engineering, and transgene‐free editing. These capabilities are advancing crop improvement not only in annual species but also in more complex systems such as polyploids, undomesticated wild relatives, and species with long generation times. At the same time, multiplex editing presents technical challenges, including complex construct design and the need for robust, scalable mutation detection. We discuss current toolkits and recent innovations in vector architecture, such as promoter and scaffold engineering, that streamline workflows and enhance editing efficiency. High‐throughput sequencing technologies, including long‐read platforms, are improving the resolution of complex editing outcomes such as structural rearrangements—often missed by standard genotyping—when targeting repetitive or tandemly spaced loci. To fully realize the potential of multiplex genome engineering, there is growing demand for user‐friendly, synthetic biology‐compatible, and scalable computational workflows for gRNA design, construct assembly, and mutation analysis. Experimentally validated inducible or tissue‐specific promoters are also highly desirable for achieving spatiotemporal control. As these tools continue to evolve, multiplex CRISPR editing is poised to become a foundational technology of next‐generation crop improvement to address challenges in agriculture, sustainability, and climate resilience.

## Significance Statement

Multiplex CRISPR editing is revolutionizing plant biotechnology by enabling simultaneous, precise modification of multiple genetic targets. This review highlights recent applications in trait stacking, functional genomics, and *de novo* domestication. Ongoing advances in CRISPR reagents, vector design, high‐throughput sequencing for mutation mapping, and computational tools are enhancing efficiency and scalability. As these toolkits evolve, multiplex editing is emerging as a foundational platform for next‐generation crop improvement.

## INTRODUCTION

As a disruptive technology, CRISPR‐Cas has made gene editing accessible across all fields of life sciences. Over the past decade, more than 55,000 studies have harnessed CRISPR‐Cas tools to modify genomes, regulate gene expression, and more, in an ever‐growing number of plant species. The system's core components are remarkably simple: a Cas nuclease or its deactivated or modified form, and one or more guide RNAs (gRNAs) that target‐specific genome sites through Watson–Crick base pairing. This simplicity underpins the versatility and adaptability of CRISPR‐Cas to a myriad of applications (Wang & Doudna, 2023) (Boxes 1 and 2).

Box 1 **Summary of main points**

- Multiplex CRISPR editing enables simultaneous manipulation of multiple loci, offering a powerful approach to dissect gene families and overcome genetic redundancy.
- It accelerates polygenic trait engineering and trait stacking, especially in complex systems such as polyploid crops, wild relatives, and perennial species.
- Applications extend beyond gene knockouts to include regulatory sequence editing, epigenetic modulation, chromosomal engineering, and combinatorial genome manipulations.
- Ongoing technical advances in construct design, delivery platforms, and mutation detection methods are expanding the scalability and precision of multiplex editing.

Box 2 **Open questions**

- How can multiplex editing workflows be scaled for high‐throughput applications across diverse plant species and genotypes?
- What strategies can minimize somatic chimerism and enhance the early recovery of homozygous or biallelic edits?
- Can multiplex systems be programmed for spatiotemporal control and combinatorial logic in gene regulation?
- What are the practical limits of multiplex editing in achieving consistent and effective outcomes across target sites?
- How can AI, machine learning, and large language models be leveraged to design, predict, and interpret multiplex editing outcomes?

However, multiplex editing remains relatively underexplored in plant sciences, with fewer than 170 papers adopting this approach at the time of this writing, representing only a small fraction (<3%) of the overall CRISPR‐related plant research published to date. Given the polygenic nature of many agronomic traits and the increasingly sophisticated plant design strategies for combinatorial trait engineering, multiplex editing is essential to fully realize the potential of CRISPR in next‐generation crop improvement (Box 1) (Fagny & Austerlitz, 2021; Gilbertson et al., 2025; Shelake et al., 2022).

Paradoxically, naturally evolved CRISPR systems are adept at multiplex editing in bacteria and archaea. All known CRISPR loci possess arrays of spacers acquired from invading organisms over time, which are deployed through highly effective parallel processing to confer adaptive immunity against subsequent invasions (Mojica et al., 2005). Repurposing CRISPR‐Cas for multiplex editing in eukaryotes, including plants, requires the construction of multiple gRNA expression cassettes and/or artificial CRISPR arrays (Cong et al., 2013; Lowder et al., 2015). However, the repeating scaffolds and other sequence elements in these systems present potential genetic engineering challenges, including construct assembly and genetic stability in both bacterial intermediates (*E. coli* and *Agrobacterium*) and the eventual plant hosts (Assaad et al., 1993; Bzymek & Lovett, 2001; Ma, Zhang, et al., 2015; Stuttmann et al., 2021).

In this review, we highlight recent advances in CRISPR multiplex editing, spanning applications from gene family characterization and polygenic trait engineering to higher‐order combinatorial genome modifications and chromosomal engineering. We examine current toolkits, their limitations, and emerging opportunities for innovation. We also discuss the analytical and bioinformatic challenges of detecting and interpreting mutations across multiple target sites, which are inherently more complex than single‐target editing. While we do not cover plant transformation bottlenecks or tissue culture‐independent strategies addressed in recent reviews (Brandizzi et al., 2025; Lee & Wang, 2023; Nasti & Voytas, 2021; Quiroz et al., 2024), we acknowledge their critical role in enabling multiplex applications. Continued exciting breakthroughs are expected in the application of multiplex editing for crop improvement, fueled by rapid advances in CRISPR toolkits and sequencing technologies.

## MULTIPLEX EDITING TO OVERCOME GENETIC REDUNDANCY

### Gene duplication and gene family characterization

Gene duplications and gene families are pervasive in plant genomes (Flagel & Wendel, 2009). The functional redundancy of paralogous genes or gene family members—whether full, partial, or overlapping—poses a major challenge for the mechanistic dissection of causal genes underlying traits of interest (Iohannes & Jackson, 2023). The precision and versatility of CRISPR editing at the gene, paralog, and family level offer significant improvements over previous gene silencing methods (e.g., antisense, RNAi, microRNA) in differentiating between specific and redundant roles of highly homologous genes.

One such example is powdery mildew resistance, where single‐gene knockouts of the *Mildew Resistance Locus O* (*MLO*) confer broad‐spectrum resistance in barley (*Hordeum vulgare*) and wheat (*Triticum aestivum*) (Angulo et al., 2023; Büschges et al., 1997; Wang, Naik, et al., 2014), but durable resistance in dicots requires multigene knockouts. In *Arabidopsis thaliana*, triple mutants (*Atmlo2 Atmlo6 Atmlo12*) were generated through successive intermutant crosses (Consonni et al., 2006). In cucumber (*Cucumis sativus* L.), multiplex knockouts of three clade V genes (*Csmlo1 Csmlo8 Csmlo11*) were necessary to achieve full resistance (Table 1) (Ma, Yang, et al., 2024). These mutants also revealed roles for calcium signaling components in powdery mildew defense, highlighting the mechanistic insights enabled by multiplex approaches (Ma, Yang, et al., 2024). The ability of a single multiplex transformation to generate both single‐ and multigene knockouts in various combinations has greatly accelerated research (Ma, Yang, et al., 2024). In annual species, novel mutations not observed in the T_0_ generation can emerge in sensitized T_1_ populations through selfing or controlled crosses (Table 1), thereby expanding the mutation spectrum without additional rounds of resource‐intensive transformation (Ma, Yang, et al., 2024; Rodríguez‐Leal et al., 2017).

**Table 1:** Representative multiplex CRISPR editing applications in plants.

Species	Trait(s)	Target #	Cas protein	gRNA #	gRNA architecture	Efficiency	Detection	Cross	Transgene‐free	References
Knockout										
*Arabidopsis thaliana*	Cell wall	3 genes	Cas9	3–4	Individual Pol III	Not provided	Sanger	Selfing	Yes	Zhang et al. (2020)
*Arabidopsis thaliana*	n/a	12 genes	Cas9	24	Individual Pol III	0–94%	Sanger	Selfing	Yes (some)	Stuttmann et al. (2021)
*Arabidopsis thaliana*	Flowering	3 genes	Cas12a	6	cRNA array (Pol II)	0–25%	Sanger, WGS	Selfing	Unknown	Jordan et al. (2021)
*Arabidopsis thaliana*	Cell wall	3 genes	Cas9	4–5	Individual Pol III, tRNA (Pol III)	Not provided	Sanger	Selfing	Unknown	Zhang et al. (2021)
*Arabidopsis thaliana*	Organ ablation	repeats	Cas9	2	Individual Pol III	Up to 89%	Amp‐seq	Selfing	Unknown	Schindele et al. (2022)
*Arabidopsis thaliana*	Growth	8 genes	Cas9	2–8	Individual Pol III, tRNA (Pol III)	0–93%	Amp‐seq	Selfing, intercross	Yes	Angulo et al. (2023)
*Arabidopsis thaliana*	Cell ablation	repeats	Cas9	1–2	Individual Pol III	Not provided	Fluorescence imaging	Selfing	Unknown	Gehrke et al. (2023)
*Citrus maxima*	Disease resistance	1 gene, 1 promoter	Cas12a, nCas9‐APOBEC1	2	tRNA (Pol III), ribozyme (Pol III)	2% transgene‐free	Sanger, WGS		Yes	Huang et al. (2023)
*Citrus sinensis*	Disease resistance	1 gene	Cas12a	3	Direct synthesis	100%	Sanger, WGS		Yes	Su et al. (2024)
*Cucumis sativus*	Disease resistance	3 genes	Cas9	3	tRNA (Pol II)	0–100%	Amp‐seq	Selfing	Yes	Ma, Yang, et al. (2024)
*Glycine max*	Nodulation	>102 genes	Cas9	1	Individual Pol III	49% overall	Sanger	Selfing	Yes (some)	Bai et al. (2020)
*Glycine max*	Oligosaccharides	2 genes	Cas9	4	tRNA (Pol III)	33–83%	Sanger	Selfing	Yes	Cao et al. (2022)
*Hordeum vulgare*	Resistant starch	7 genes	Cas9	7	Individual Pol III	0–74%	PCR	Selfing	Unknown	Yang et al. (2024)
*Oryza sativa*	Disease resistance, male sterility	3 genes	Cas9	3	Individual Pol III	Not provided	Sanger	Selfing	Yes	Li et al. (2019)
*Oryza sativa*	Resistant starch	4 genes	Cas9	4	tRNA (Pol III)	25–88%	Amp‐seq	Selfing	No	Biswas et al. (2023)
*Oryza sativa FXZ*	Growth	12 genes	Cas9	6 or 12	Individual Pol III	68–100%	Amp‐seq, WGS	Selfing, intercross	Yes	Wei et al. (2024)
*Populus davidiana × bolleana*	Flowering	2 genes	nCas9‐APOBEC3A	2	Individual Pol III	2% transgene‐free, 7% biallelic CEN1	Sanger, WGS		Yes	Wu et al. (2025)
*Populus tremula × alba*	Lignin	2 genes	Cas9	1 (2 sets)	Individual Pol III	0–62%	Sanger		No	de Vries et al. (2021)
*Populus tremula × alba*	Trichome	3 genes (8 alleles)	Cas9	1	Individual Pol III	97–100%	Amp‐seq		No	Bewg et al. (2022)
*Populus tremula × alba*	Reproduction	2–6 genes	Cas9	1–3	Individual Pol III	100%	Amp‐seq		No	Ortega et al. (2023)
*Populus tremula × alba*	Lignin	3 genes	nCas9‐APOBEC3A	2	Individual Pol III	7% transgene‐free, 0% biallelic CCoAOMT1	Sanger, WGS		Yes	Hoengenaert et al. (2025)
*Populus trichocarpa*	Lignin	10 genes	Cas9	3–6 (7 sets)	tRNA (Pol III)	0–100%	Sanger, Amp‐seq		No	Sulis et al. (2023)
*Solanum lycopersicum*	Multiple traits	8 genes	Cas12a, nCas9‐APOBEC3A	2	tRNA (Pol III), ribozyme (Pol II)	8–42% transgene‐free	Sanger, WGS		Yes	Huang et al. (2023)
*Solanum lycopersicum*	Fruit color	3 genes	Cas9	6	tRNA (Pol III)	8–92%	Sanger	BC, selfing	Yes	Yang et al. (2023)
*Zea mays*	Yield, drought tolerance	48 genes	Cas9	12 (4 sets)	Individual Pol III	73% overall	Amp‐seq	Selfing, intercross	Yes (some)	Lorenzo et al. (2023)
Editing of tandemly arrayed genes										
*Arabidopsis thaliana*	Disease resistance	4 TAGs	Cas9	2	Individual Pol III	Not provided	Sanger	Selfing	No	Shen et al. (2019)
*Arabidopsis thaliana*	Reproduction	7 TAGs	Cas9	2 or 6	Individual Pol III	Not provided	Sanger	Selfing	Yes	Zhong et al. (2019)
*Arabidopsis thaliana*	n/a	Mulittags of 2–6	Cas9	2 (multisets)	Individual Pol III	0–78% TAG deletions	PCR, Sanger	Selfing	Unknown	Liu et al. (2023)
*Oryza sativa*	Disease resistance	2 genes	Cas9	4	Individual Pol III	Not provided	Sanger		Unknown	Shen et al. (2022)
*Populus tremula × alba*	n/a	7 TAGs	Cas9	1	Individual Pol III	98–100%	PCR, Amp‐seq, Capture‐seq		No	Chen et al. (2023)
Polyploid engineering										
*Dendrocalamus latiflorus*	Height	3 genes	Cas9	1	Individual Pol III	40%	Sanger		No	Ye et al. (2020)
*Musa spp*	Virus resistance	3 genes	Cas9	3	Individual Pol III	70–85%	Sanger		No	Tripathi et al. (2019)
*Musa acuminata*	Herbicide tolerance	2 genes	nCas9‐APOBEC1	1–2	Individual Pol III	45–53% (1–3% transgene free)	Sanger, WGS		Yes	Van den Broeck et al. (2025)
*Nicotiana benthamiana*	Therapeutic proteins	6 genes	Cas9	3–7	tRNA (Pol III)	0–83%	Sanger	Selfing	Unknown	Jansing et al. (2019)
*Nicotiana benthamiana*	n/a	9 genes	Cas9	10	Individual Pol III	96.50%	Sanger		No	Stuttmann et al. (2021)
*Oryza alta*	Domestication	Many	Cas9	2–8 (multisets)	Individual Pol III	Not provided	Sanger		No	Yu et al. (2021)
*Saccharum spp. hybrid*	Chlorophyll	59 alleles	Cas9	2	Individual Pol III	14–20%	Sanger and Amp‐seq		No	Eid et al. (2021)
*Triticum aestivum*	Allergen reduction	>17 targets	Cas9	7	tRNA (Pol III)	21–100%	Sanger	Selfing	Yes	Camerlengo et al. (2020)
*Triticum aestivum*	Resistant starch	3 genes	Cas9	1 (2 sets)	Individual Pol III	33–67%	Sanger	Selfing	Yes	Li et al. (2021)
*Triticum aestivum*	Multiple traits	6 genes	Cas9	2–5	tRNA (Pol II)	18–75%	PCR, Sanger	Selfing, embryo rescue	Yes	Luo et al. (2021)
*Triticum aestivum*	Allergen reduction	>32 targets	Cas9	2 or 4	Individual Pol III	0–100% (34% overall)	Sanger and Amp‐seq	Selfing	Yes	Sánchez‐León et al. (2024)
*Triticum aestivum*	Allergen reduction	>89 targets	Cas9	1 (7 sets)	Csy4 (Pol II)	Not provided	PCR, Amp‐seq, WGS	Selfing	Yes	Yu et al. (2024)
Transcriptional regulation (including combinatorial editing)										
*Arabidopsis thaliana*	Flowering time	1 promoter (methylation)	dCas9	3	Individual Pol III	25% (DNA methylation)	RT‐qPCR, bisulfite PCR sequencing	Selfing	Yes	Ghoshal et al. (2021)
*Arabidopsis thaliana*	Rapid breeding	1 promoter, 4 genes	Cas9, nCas9‐APOBEC3A	2	Individual Pol III	~3–65%	Amp‐seq	Selfing	Yes	Pan et al. (2022)
*Arabidopsis thaliana*	Flowering	1 promoter	dCas9	2	Individual Pol III	80% (activation)	RT‐qPCR	Selfing	No	Zinselmeier et al. (2024)
*Arabidopsis thaliana*	Root flavonoids	6 promoters	dCas9	6–12	Individual Pol III	Not provided	Fluorescence imaging	Selfing	No	Houbaert et al. (2025)
*Oryza sativa*	Grain quality	1 promoter +5’‐UTR	Cas9	1–2	Individual Pol III	Not provided	PCR, Sanger	Selfing	Yes	Zeng et al. (2020)
*Oryza sativa*	Regeneration	1 promoter, 2 genes	Cas9	2	tRNA (Pol II)	37–88%	RT‐qPCR, Amp‐seq		No	Pan et al. (2022)
*Oryza sativa*	Yield	1 promoter + UTRs	Cas9	2–4 (39 sets)	Individual Pol III	19%	PCR, Sanger	Selfing	Yes	Song et al. (2022)
*Populus tremula × alba*	Regeneration	2 promoters, 1 gene	Cas9	2	Individual Pol III	50–80%	RT‐qPCR, Amp‐seq		No	Pan et al. (2022)
*Solanum lycopersicum*	Fruit traits	2 promoters	Cas9	8	Individual Pol III	Not provided	PCR, Sanger, WGS	BC, selfing	Yes	Rodríguez‐Leal et al. (2017)
*Solanum lycopersicum*	Productivity	2 promoters	Cas9	8	Individual Pol III	Not provided	PCR, Sanger	BC, selfing	Yes	Wang et al. (2021)
*Solanum lycopersicum, Physalis grisea*	Branching	1 promoter	Cas9	6–8	Individual Pol III	Not provided	PCR, Sanger, WGS	BC, selfing	Yes	Hendelman et al. (2021)
*Zea mays*	Grain yield	2 promoters	Cas9	9	Unspecified	Not provided	PCR, Sanger	BC, selfing	Yes	Liu et al. (2021)
Chromosome engineering										
*Arabidopsis thaliana*	Chr translocation	2 targets	Cas9	2	Individual Pol III	0.01–0.05%	PCR, Amp‐seq, FISH	Selfing	Yes	Beying et al. (2020)
*Arabidopsis thaliana*	Chr inversion	2 targets	Cas9	2	Individual Pol III	0.50%	PCR, Sanger	Selfing	Unknown	Schmidt et al. (2020)
*Arabidopsis thaliana*	Chr inversion	2 targets	Cas9	2	Individual Pol III	Not provided	PCR, Sanger, FISH	Selfing	Yes	Rönspies et al. (2022)
*Arabidopsis thaliana*	Chr inversion		Cas9	2	Individual Pol III	0.06–0.25%	PCR, Sanger, FISH	Selfing	Yes	Khosravi et al. (2025)
*Zea mays*	Chr inversion	2 targets	Cas9	2	Direct synthesis	0.13%	Amp‐seq, Bionano	Selfing	No	Schwartz et al. (2020)

Studies focused on tool development, reporter genes, transient expression, or lacking whole plant regeneration were not included.

Amp‐seq, amplicon sequencing; BC, backcross; Chr, chromosome; FISH, fluorescence in situ hybridization; TAG, tandemly arrayed genes; WGS, whole‐genome sequencing.

In hybrid poplar (*Populus tremula* × *P. alba* INRA 717‐1B4), a single gRNA targeting a conserved sequence among *MYB* transcription factor genes successfully edited three known paralogs (*PtaMYB186*, *PtaMYB138*, and *PtaMYB38*), along with two additional tandem duplicates in one of the subgenomes that were only identified after sequencing the transformation genotype (Bewg et al., 2022; Zhou et al., 2023). This underscores the importance of accurate genome annotation for interpreting copy number variation and multiplex editing outcomes. Editing all eight alleles resulted in glabrous phenotypes and uncovered a link between triterpene biosynthesis and non‐glandular trichomes in poplar (Bewg et al., 2022). This study also demonstrated that multiplex editing of closely linked genes can induce large genomic deletions (~29–62 Kb). These structural variants are likely underestimated by standard indel‐focused genotyping methods.

In another example, multiplex editing clarified the role of caffeoyl shikimate esterase (CSE) in lignin biosynthesis. While single knockouts of *CSE* paralogs in poplar had no effect, double mutants showed reduced lignin content and altered composition (de Vries et al., 2021). Mutant characterization further revealed partial redundancy and broader substrate specificity, which were further supported by *in vitro* enzyme kinetics (de Vries et al., 2021). This work highlights the value of generating both single and multiplex mutant lines to disentangle functional redundancy and specificity.

Multiplex editing is also valuable for gene family investigation in *A. thaliana*, despite the availability of extensive mutant collections from chemical and insertional mutagenesis (Kim et al., 2006; Krysan et al., 1999). Generating higher‐order mutants by crossing is time‐consuming and often impractical for closely linked genes. Furthermore, traditional mutants frequently harbor secondary mutations (Carrère et al., 2024; Raabe et al., 2024), which complicate data interpretation. CRISPR multiplex editing enables the direct generation of complex mutant combinations in a single generation, streamlining functional analysis. For example, Zhang et al. (2020) used six gRNAs in two constructs to target three *GLCAT14* genes encoding β‐glucuronosyltransferases and successfully generated single, double, and triple knockout mutants in the T_1_ generation. Mutant characterization revealed redundant roles of GLCATs in arabinogalactan‐protein (AGP) glycosylation, with pleiotropic effects on seed germination, tip growth, pollen function, and seed coat mucilage (Zhang et al., 2020). Similarly, multiplex editing of five hydroxyproline‐*O*‐galactosyltransferase genes produced triple and quintuple mutants with additive effects on AGP glycosylation, overall growth, root development, and reproduction (Zhang et al., 2021). These studies demonstrate how multiplex editing accelerates the dissection of gene family function and reveals phenotypic complexity masked by redundancy.

Other recent applications of multiplex editing for investigating functional redundancy include the raffinose synthase family in soybean (Cao et al., 2022) and the growth‐regulating factor (GRF) family in *A. thaliana* (Angulo et al., 2023). The GRF study represented the cumulative progress of two undergraduate laboratory course sessions and one undergraduate research project, showcasing the power of CRISPR in experiential learning for life science undergraduate education.

### Tandemly arrayed genes

Tandemly arrayed genes (TAGs) derived from tandem duplications constitute a substantial portion of plant genomes (Rizzon et al., 2006; Yu et al., 2015). Their high sequence similarity and functional redundancy often necessitate multigene knockouts for effective functional analysis. TAGs can undergo homologous or unequal recombination, leading to either functional diversification or degeneration (Hanada et al., 2008; Otto et al., 2022). Disentangling the functional fates of TAGs is particularly challenging using conventional genetic approaches due to low recombination rates between closely linked loci (Jander & Barth, 2007). Consequently, functional studies of TAGs remain limited.

Multiplex CRISPR editing has enabled functional dissection of TAGs in ways that were previously impractical. In *A. thaliana*, sequential multiplex transformations targeting the *AtLURE1* family revealed that these cysteine‐rich LURE1 peptides promote conspecific pollen tube attraction and reproductive isolation, rather than simply impeding interspecific fertilization (Zhong et al., 2019). Although effective, the need for multiple transformation rounds highlights the limitation of this approach in species with long generation times.

**Figure 1:**
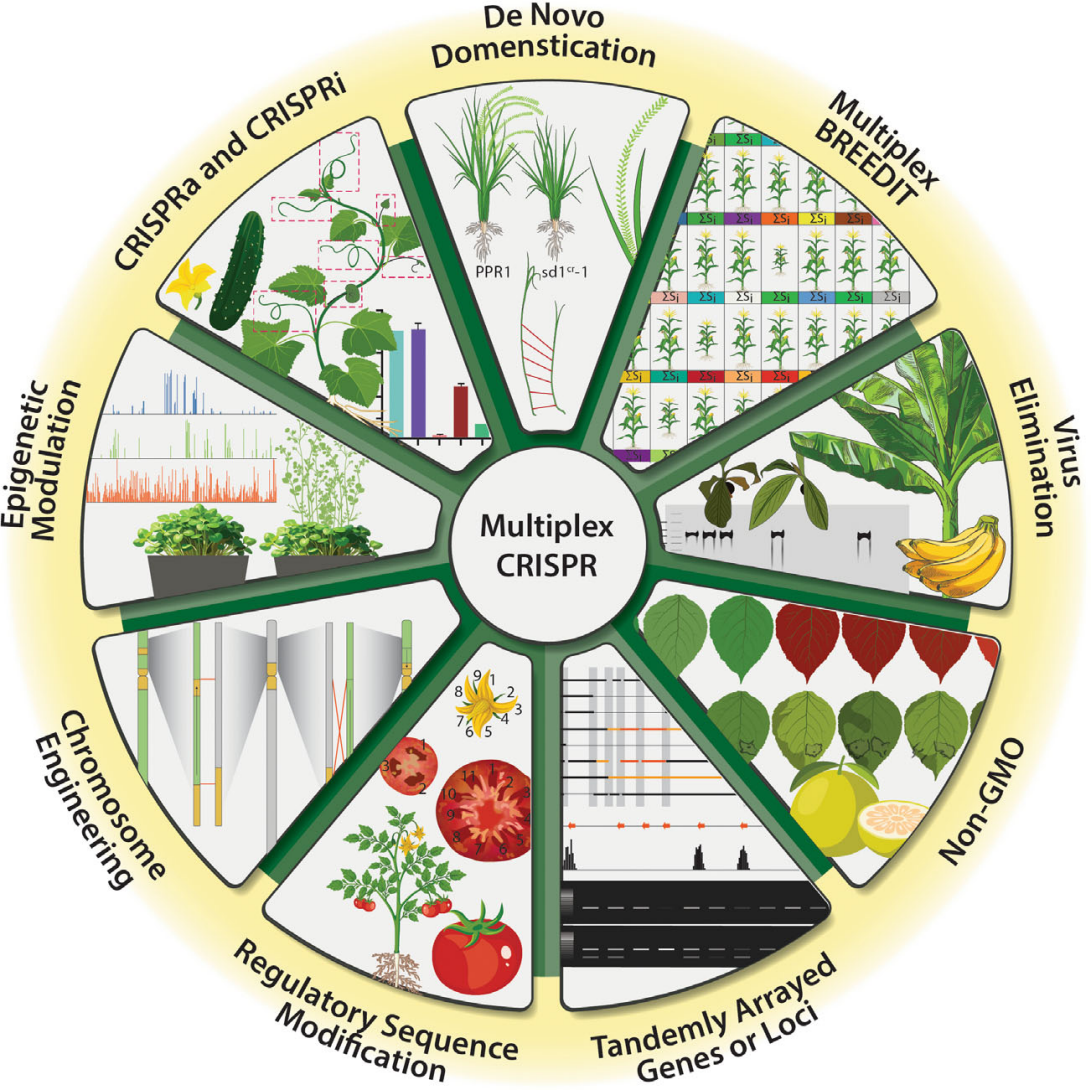
Applications of multiplex CRISPR genome editing in plants. This circular diagram illustrates key applications of multiplex CRISPR in plant systems: (1) CRISPR activation and interference (CRISPRa and CRISPRi) for gene regulation; (2) *de novo* domestication of wild species; (3) multiplex‐assisted breeding strategies; (4) virus elimination in crops such as banana; (5) development of non‐GMO varieties; (6) editing of tandemly arrayed genes or loci; (7) modification of regulatory sequences; (8) chromosome engineering; and (9) epigenetic modulation. These applications underscore the versatility of multiplex genome editing in advancing both basic research and crop improvement. Sources adapted with permission or under Creative Commons licenses (CC BY/CC BY 4.0).

In contrast, a single multiplex transformation in hybrid poplar successfully disrupted all seven *Nucleoredoxin1* genes within a ~100 kb TAG cluster using one gRNA (Chen et al., 2023). This generated a wide spectrum of mutations, from small indels to large deletions and complex rearrangements, including translocations, inversions, and multi‐fragment fusions, many of which escaped detection by standard PCR or amplicon sequencing (Chen et al., 2023). Capture sequencing with *de novo* assembly resolved some of these structural variants (Figure 1, Table 1), underscoring the need for advanced genotyping strategies in TAG editing.

Similar outcomes have been reported in *A. thaliana* and rice (*Oryza sativa*), where multiplex editing of TAGs, encoding phytocytokines, metacaspases, chitinases, and disease resistance proteins led to large deletions and inversions (Table 1) (Liu et al., 2023; Shen et al., 2019; Shen et al., 2022). It should be noted that chromosomal deletions and inversions are not limited to TAGs *per se*, and can occur between closely linked genes (Liu et al., 2023) or between distant target sites on the same chromosome as directed by gRNAs (Khosravi et al., 2025). While modified PCR assays can distinguish inversions from deletions (Liu et al., 2023), more complex rearrangements remain difficult to detect. These findings highlight both the power and technical challenges of multiplex editing in TAG loci and emphasize the importance of comprehensive mutation profiling.

### Polyploid genome engineering

CRISPR genome editing is a game changer for polyploid species with inherently complex genomes. For instance, sugarcane cultivars are polyploid/aneuploid interspecific hybrids of *Saccharum officinarum* and *Saccharum spontaneum*, possessing 10–13 sets of the 10 basic chromosomes (Souza et al., 2019). Similarly, woody bamboos encompass over 1500 species of tetraploids or hexaploids with 12 chromosomes (Ma, Liu, et al., 2024). As such, multiplex (multiallele) editing is essential in polyploid species, even for single‐gene targets, to obtain null mutants.

In sugarcane, targeting a chlorophyll biosynthetic gene with two gRNAs resulted in mutations across 49 of 59 alleles, demonstrating the feasibility of multiplex editing to address genetic redundancy in highly polyploid crops (Eid et al., 2021). Similarly, in hexaploid Ma bamboo (*Dendrocalamus latiflorus* Munro), multiplex editing of a gibberellin‐responsive gene produced null mutants with increased plant height (Ye et al., 2020).

In wheat, multiplex editing has been applied to distinct gene families regulating gluten‐related traits (Table 1). Seven gRNAs were used to edit WTAI (wheat tetrameric α‐amylase/trypsin inhibitor) subunits CM3 and CM16 in an elite durum wheat cultivar to reduce allergens associated with baker's asthma (Camerlengo et al., 2020). In separate studies, multiple gRNAs targeted γ‐ and ω‐gliadin gene clusters in hexaploid bread wheat, achieving 64–97% reductions in gliadin content in homozygous, transgene‐free lines (Sánchez‐León et al., 2024; Yu et al., 2024). Crosses with previously edited α‐gliadin‐deficient lines (Sánchez‐León et al., 2018) further reduced total gluten levels (Sánchez‐León et al., 2024), offering a promising path toward gluten‐free wheat. The complications associated with TAG editing, as discussed in the previous section, are exacerbated in polyploid crops lacking high‐quality reference genomes, underscoring universal challenges for multiplex editing applications.

## QUANTITATIVE TRAIT ENGINEERING AND STACKING

### Speedy polygenic trait engineering

Plant traits are rarely controlled by single genes, making multiplex gene editing a powerful tool for accelerating trait improvement. In tomato (*Solanum lycopersicum*), fruit color diversity was engineered by simultaneously editing three genes, *PSY1* (*phytoene synthase 1*), *MYB12*, and *SGR1* (*STAYGREEN 1*), using two gRNAs per gene (Yang et al., 2023). The green‐fruited *psy1 myb12 sgr1* triple mutant was backcrossed with wild type, followed by selfing of transgene‐free F1 plants to generate a segregating population with novel fruit colors in less than a year (Yang et al., 2023).

In cereals, dietary fiber and resistant starch (RS) content are influenced by genes encoding multiple starch synthases and starch branching enzymes. Multiplex editing of *SBE* genes in wheat and rice significantly increased amylose and RS levels, with stronger effects in higher‐order mutants (Biswas et al., 2023; Li et al., 2021). In barley, targeting four *SS* and three *SBE* genes revealed combinatorial effects on RS content and grain traits (Yang et al., 2024). While full knockouts were not recovered, polygenic mutants showed enhanced RS and, in some cases, partial rescue of yield penalties (Yang et al., 2024). This suggests that multiplex editing strategies can be used to create and select lines with improved dietary fiber quality while minimizing negative effects on grain production.

In *Nicotiana benthamiana*, two *β‐1,2‐xylosyltransferase* genes and four *α‐1,3‐fucosyltransferase* genes were simultaneously edited to modify N‐glycosylation patterns in plant‐derived therapeutic proteins (Table 1). The resulting sextuple mutants produced recombinant glycoproteins lacking plant‐specific α‐1,3‐fucose and β‐1,2‐xylose residues, with antibody‐binding behavior comparable to that from mammalian systems (Jansing et al., 2019). The rapid modifications enabled by CRISPR multiplex editing hold promise for overcoming limitations in developing plant‐based glycoproteins for therapeutic applications.

In black poplar (*Populus trichocarpa*), a predictive modeling approach was used to evaluate over 69,000 possible combinations of manipulations across 21 lignin biosynthetic genes aimed at improving wood properties (Sulis et al., 2023). Seven multiplex editing strategies, each targeting three to six genes, were selected for *in planta* testing. These strategies were predicted to reduce total lignin content and increase the syringyl‐to‐guaiacyl lignin ratio without negatively affecting plant growth. Although complete knockouts of all target genes were not achieved, some edited lines exhibited improved cellulose‐to‐lignin ratios and enhanced pulping efficiency (Sulis et al., 2023). This work highlights the potential of integrating computational pathway modeling with multiplex editing for complex trait optimization.

In soybean, a CRISPR library targeting 102 genes and their paralogs enabled pooled transformation and trait screening (Bai et al., 2020). This approach uncovered synergistic mutation combinations affecting nodulation without compromising growth (Bai et al., 2020). For species with low transformation rates, this library‐based strategy offers both scalability and a streamlined path for trait discovery, especially when primary transformants can be crossed to increase the mutation diversity. However, its application remains challenging in long‐lived perennials or clonally propagated species due to difficulties in maintaining and propagating mutant lines.

### Facile trait stacking and de novo domestication

Multiplex gene editing is particularly well‐suited for stacking desirable alleles spanning multiple traits (Box 1). In rice, thermosensitive male sterility and disease resistance were simultaneously engineered into a breeding line (Li et al., 2019). Transgene‐free, homozygous triple mutants were obtained in the T_1_ generation to accelerate hybrid rice development (Table 1) (Li et al., 2019). Similarly, editing six agronomic genes in an elite wheat variety enabled targeted mutagenesis at up to 15 loci (Luo et al., 2021). This approach allowed rapid pyramiding of favorable alleles controlling multiple traits in hexaploid wheat within a single year, representing a substantial time savings over traditional breeding methods (Luo et al., 2021).

In maize, the BREEDIT pipeline combines multiplex genome editing with conventional crosses to generate over 1000 edited lines targeting 48 growth‐related genes using four 12‐plex constructs (Lorenzo et al., 2023). This approach enabled allele stacking, the recovery of fixed mutations, and the generation of new edits through strategic crosses (Figure 1). Some lines exhibited significant increases in leaf size and drought tolerance (Lorenzo et al., 2023). A similar strategy was applied to a new rice cultivar, FXZ, which has high storage quality and disease resistance but suffers from slow maturation and low adaptability (Wei et al., 2024). Multiplex editing of 12 agronomic genes and rational crosses produced a segregating population with diverse phenotypes. Selected plants exhibited improved early heading and plant architecture without compromising yield or disease resistance (Wei et al., 2024).

Multiplex editing also enables *de novo* domestication of crop wild relatives by directly targeting domestication genes while preserving beneficial traits to expand gene pools (Kumar et al., 2022; Lemmon et al., 2018; Wolter et al., 2019; Zsögön et al., 2018). Yu et al. (2021) developed a strategic roadmap integrating genome sequencing, efficient transformation, and multiplex editing for *de novo* domestication of wild allotetraploid rice (*Oryza alta*) (Figure 1). Using a combination of loss‐of‐function (via single or multiplex mutagenesis) and gain‐of‐function (via base editing) approaches, the team achieved rapid improvements in traits such as seed shattering, grain size, and heading date, while retaining stress resilience from wild germplasm (Yu et al., 2021). This strategy offers a fast‐track route to the development of new crop varieties for enhanced resistance to biotic and abiotic stresses under changing climate conditions.

In long‐lived species like poplar, multiplex editing can circumvent long generation times to accelerate research discovery. An *in vitro* flowering system was developed by knocking out two paralogs of *CEN* (*Centroradialis*), a key floral repressor (Ortega et al., 2023). This fast‐track system is more efficient than heat‐inducible *FT* (*FLOWERING LOCUS T*) methods (Hoenicka et al., 2016) and allows flexible allele pyramiding for floral trait investigation. For instance, simultaneous editing of *ARR17* (a type‐A response regulator and the main sex regulator) (Müller et al., 2020) and *CEN* genes induced male flower development *in vitro*. Allele stacking with edits in trichome‐regulating *MYBs* yielded glabrous female mutants with hairless seeds (Ortega et al., 2023). These findings revealed shared regulatory pathways between vegetative and reproductive (seed) trichomes and offer promising targets for genetic containment and allergen reduction in urban and plantation forestry.

### Eradication of endogenous viruses

Multiplex editing has also opened new avenues for the eradication of endogenous viruses, a breakthrough first demonstrated in porcine cells to inactivate porcine endogenous retroviruses, which are major obstacles in xenotransplantation (Yang et al., 2015). Similar endogenous viral elements in crops, particularly those propagated vegetatively, can severely disrupt breeding programs (Staginnus & Richert‐Pöggeler, 2006). A notable example is the endogenous banana streak virus (eBSV), which has triggered recent outbreaks of banana streak mosaic disease. These outbreaks originated from breeding lines and micropropagated hybrids of *Musa accuminata* (A genome) and *Musa balbisiana* (B genome), the latter being a known reservoir of integrated eBSV sequences (Gayral et al., 2008). This genomic integration has significantly limited the use of *Musa balbisiana* in banana and plantain breeding. Tripathi et al. (2019) used CRISPR multiplex editing to target three ORFs of eBSV in plantain cultivar Gonja Manjaya (AAB genome) and achieved high mutation rates and symptom suppression in several edited lines (Figure 1). DNA analysis indicated that simultaneous disruption of all three ORFs was key to effective virus inactivation (Tripathi et al., 2019). While long‐term monitoring is still needed, this study demonstrates a promising strategy for generating virus‐free lines in primary transformants of vegetatively propagated crops.

## BEYOND GENE KNOCKOUTS

### Regulatory sequence editing

Multiplex promoter editing, first demonstrated in tomato by Rodríguez‐Leal et al. (2017), is a powerful strategy for fine‐tuning gene expression without prior knowledge of *cis* regulatory elements. Random mutagenesis, including large deletions, at multiple upstream target sites can produce wide‐ranging effects on gene expression, extending beyond traditional gene knockout or activation. When applied to regulatory genes such as transcription factors (TFs), promoter edits can influence not only the expression of the specific TF but also its upstream regulators, downstream targets, and other interacting partners. These cascading effects may include changes in TF dosage, timing, and spatial patterns of expression and can result in complex phenotypic outcomes that are not necessarily correlated with the expression level of the target gene alone. When combined with selfing or backcrossing, this ‘multiplex mutagenesis drive system’ can rapidly generate large mutant populations with a continuum of genetic variation for quantitative trait engineering (Figure 1) (Hendelman et al., 2021; Liu et al., 2021; Rodríguez‐Leal et al., 2017; Wang et al., 2021). The strategy is also applicable to UTRs and introns that may harbor *cis* regulatory elements (Table 1) (Song et al., 2022; Zeng et al., 2020).

Multiplex editing of regulatory sequences has yielded novel or beneficial traits such as modified tomato fruit size (Aguirre et al., 2023; Rodríguez‐Leal et al., 2017), multiple ear traits linked to increased maize grain yield (Liu et al., 2021), improved rice grain quality with desirable amylose content (Zeng et al., 2020), as well as increased tiller number and panicle size (Song et al., 2022). Many of these traits have been difficult to stack through conventional breeding due to time constraints, tradeoffs, and linkage drag. Multiplex editing of regulatory elements offers an efficient means to overcome these limitations and disentangle complex trait interactions.

Beyond trait improvement, this approach has advanced our understanding of key developmental circuits. For example, it has been used to dissect the Clavata–Wuschel (CLV‐WUS) pathway regulating stem cell proliferation in tomato (Rodriguez‐Leal et al., 2019; Rodríguez‐Leal et al., 2017; Wang et al., 2021) and maize (Liu et al., 2021), and to explore epistatic relationships among the novel fruit size alleles (Aguirre et al., 2023). An allelic series generated through multiplex editing revealed previously unknown pleiotropic roles of WOX9 (WUS‐related homeobox 9) during vegetative and reproductive development conserved across tomato, groundcherry, and Arabidopsis (Hendelman et al., 2021). Notably, distinct pleiotropic functions were associated with specific *cis* regulatory regions (Hendelman et al., 2021), underscoring the power of regulatory sequence editing to partition gene function in ways not possible with null mutations.

In rice, editing of *IPA1* (*Ideal Plant Architecture 1*) regulatory elements led to the discovery of a 54 bp promoter deletion that underlies the tradeoff between tiller and panicle number (Song et al., 2022). Mechanistic studies identified a motif within this region as the binding site for An‐1, a basic helix–loop–helix transcription factor that represses *IPA1* expression in panicles and roots (Luo et al., 2013; Song et al., 2022). These examples illustrate how multiplex editing of regulatory sequences enables both precision trait engineering and mechanistic dissection of complex gene networks.

### Epigenetic and transcriptional regulation

The multiplex CRISPR system has been adapted for transcriptional regulation and epigenetic modification without altering DNA sequences (Box 1). Recent reviews have summarized the development of effector domains and strategies for their recruitment in plant systems (Gardiner et al., 2022; McCarty et al., 2020). The core design uses a catalytically dead Cas9 (dCas9) for site‐specific targeting via multiple gRNAs, with effectors either fused directly to dCas9 or recruited through systems such as SunTag (Tanenbaum et al., 2014), MoonTag (Casas‐Mollano et al., 2023), or modified gRNA scaffolds (Konermann et al., 2015).

Effectors for epigenetic modifications include catalytic domains from DNA methyltransferases (Ghoshal et al., 2021), demethylases (Gallego‐Bartolomé et al., 2018), and histone acetyltransferases of bacterial, mammalian, or plant origin (Lee et al., 2019; Roca Paixão et al., 2019; Selma et al., 2019; Wang, Liu, et al., 2024). These tools modulate promoter accessibility and can fine‐tune gene expression with potentially fewer off‐target effects than direct transcriptional activation (Gardiner et al., 2022). For example, DNA demethylation at the *FLOWERING WAGENINGEN* (*FWA*) promoter using a single gRNA significantly upregulated *FWA* expression and delayed flowering in *Arabidopsis* (Gallego‐Bartolomé et al., 2018). In contrast, targeted methylation directed by multiple gRNAs led to *FWA* silencing and early flowering (Ghoshal et al., 2021) (Figure 1). Importantly, the modified DNA methylation states and associated phenotypes were heritable, even in transgene‐free progeny (Gallego‐Bartolomé et al., 2018; Ghoshal et al., 2021). Despite these promising findings, further optimization is needed to enhance efficiency and specificity, particularly with regard to CG, CHG, and CHH contexts, and to ensure long‐term stability of these modifications (Gardiner et al., 2022).

CRISPR‐based transcriptional activation (CRISPRa) and interference (CRISPRi) use effector domains to modulate gene expression without editing DNA. Advanced CRISPRa systems combine dCas9‐activator fusions with MS2 aptamer‐containing gRNA scaffolds and/or SunTag‐based multimeric recruitment (Pan et al., 2021; Selma et al., 2022). Multiplex CRISPRa targeting up to six steps in the flavonoid biosynthetic pathway can selectively induce different flavonoid classes (Selma et al., 2022), including in a cell‐type‐specific manner (Houbaert et al., 2025). A recent study used inducible multiplex CRISPRa for large‐scale screening of morphogenic regulators to enhance plant regeneration in alfalfa (*Medicago sativa*), strawberry (*Fragaria vesca*), and sheepgrass (*Leymus chinensis*) (Zhang et al., 2024). Similarly, a copper‐inducible system enabled tunable activation of metabolic genes in *Nicotiana benthamiana* (Garcia‐Perez et al., 2022).

MoonTag, with its peptide‐nanobody design, has shown improved performance over SunTag in stable transformants (Casas‐Mollano et al., 2023). Replacing dCas9 with dCas12b or the near‐PAMless dSpRY variant expands the targeting space (Pan et al., 2021). CRISPR‐Combo enables simultaneous editing and activation (Pan et al., 2022) by using Cas9 with long gRNAs (18–20 nt) for cleavage and short gRNAs (≤16 nt) with modified scaffolds for binding without cleavage (Kiani et al., 2015). This allows a single Cas enzyme—whether Cas9, PAMless SpRY, or base editor derivatives—to perform multiplex editing and gene activation across species (Pan et al., 2022). Applications included editing agronomic genes while activating *FT* for rapid generation cycling in *Arabidopsis* or morphogenic regulators for accelerated regeneration in poplar and rice (Table 1) (Pan et al., 2022).

Another strategy uses engineered gRNA scaffolds to recruit cytidine and adenosine deaminases via their cognate binding proteins, enabling concurrent C‐to‐T and A‐to‐G base editing of different genes using a single dCas9 (Li et al., 2020). When integrated with CRISPR‐Combo, this design can support higher‐order multiplexing for simultaneous knockout, gain‐of‐function base substitutions, and transcriptional modulation (activation or silencing) at distinct loci.

Recent screening of plant‐ and pathogen‐derived activator domains identified several that outperform VP64 in SunTag‐ and MoonTag‐based multiplex CRISPRa systems in *Arabidopsis* and *Setaria* (Zinselmeier et al., 2024). In contrast, CRISPRi remains less explored in plants (Lowder et al., 2015; Piatek et al., 2015), likely due to the availability of RNAi and artificial miRNA tools. Beyond the commonly used SRDX (Superman Repression Domain X) domain, recent studies have shown effective repression using EAR motifs such as DLN114 and its fusions with SRDX (Xu et al., 2023) and ZAT10 (Khan et al., 2025). A novel repressor domain from the cucumber (*Cucumis sativus*) non‐canonical histone acetyltransferase TENDRIL‐LESS (CsTEN) has been fused with dCas9 to enable programmable transcriptional repression of a wide range of genes in Arabidopsis and, for the first time, in cucumber plants (Figure 1) (Wang, Liu, et al., 2024). Multiplex CRISPRi has also been used to construct reversible gene circuits for spatiotemporal regulation in plants (Khan et al., 2025).

## CHROMOSOMAL ENGINEERING

Multiplex CRISPR toolkits have been leveraged to induce targeted chromosomal rearrangements, including inversions, translocations, and arm exchanges (Box 1). In maize, simultaneous induction of double‐strand breaks (DSBs) on opposite sides of the centromere generated a 75.5 Mb pericentric inversion on chromosome 2 (Schwartz et al., 2020). In Arabidopsis, two DSBs near the telomeric ends of chromosome 2 led to a heritable ~17 Mb inversion, retaining only 2 Mb and 0.5 Mb of the original telomeric ends (Rönspies et al., 2022). Multiplex editing also enabled reversal of the 1.1 Mb heterochromatic knob (hk4S) inversion on chromosome 4—an ancient rearrangement estimated to have occurred ~5000 years ago in some Arabidopsis accessions (Schmidt et al., 2020). Crossing the rearranged Col‐0 with Ler‐1 (which lacks the inversion) restored meiotic crossovers in a previously recombination‐suppressed region (Figure 1) (Schmidt et al., 2020). Additional studies, also in Arabidopsis, demonstrated heritable megabase‐scale arm exchanges between chromosomes 1 and 2, and 1 and 5, following multiplex‐induced DSBs (Beying et al., 2020). In both cases, the chromosome‐rearranged Arabidopsis lines were morphologically indistinguishable from the wild type (Beying et al., 2020; Schmidt et al., 2020). However, restoration of meiotic recombination in otherwise suppressed regions represents a notable phenotypic outcome with potential implications for breeding and trait discovery.

A visual assay using a hemizygous betalain pigment marker was developed to help detect rare somatic crossovers in tomato. This system confirmed that multiplex‐induced DSBs can trigger a spectrum of structural changes, including crossovers, chromosomal loss, and chromothripsis‐like rearrangements (Samach et al., 2023). Notably, megabase‐scale inversions had minimal impact on global gene expression and epigenetic state in Arabidopsis (Khosravi et al., 2025). This suggests that targeted inversions could be a powerful tool for fixing beneficial haplotypes and suppressing unwanted recombination in crop breeding.

Multiplex CRISPR has also been adapted for cell‐type‐specific ablation. The CRISPR‐Kill system targets repetitive 45S ribosomal RNA (rDNA) genes to induce cell death (Table 1) (Schindele et al., 2022). When driven by tissue‐specific promoters, CRISPR‐Kill enabled selective elimination of petal cells or reduction of lateral roots in Arabidopsis (Schindele et al., 2022). Coupled with a chemically inducible system, CRISPR‐Kill also allows temporal control of cell ablation (Gehrke et al., 2023), offering new tools for developmental studies and synthetic biology.

## TRANSGENE‐FREE EDITED CROPS

### From protoplast delivery to agrobacterium transient expression

One of the biggest challenges facing gene‐edited crops is the regulatory framework governing transgenic plants, which hinders not only commercialization but also field trials essential for trait evaluation (Boerjan & Strauss, 2024; Ivanov et al., 2025). This emphasizes the need for transgene‐free CRISPR‐Cas technologies. DNA‐free editing was first demonstrated through direct delivery of preassembled Cas9–gRNA ribonucleoproteins (RNPs) into protoplasts for plant regeneration (Woo et al., 2015). This method was later adapted to generate canker‐resistant citrus (*Citrus sinensis*) via Cas12a single or multiplex editing of the susceptibility gene *LOB1* (lateral organ boundaries) in embryogenic protoplasts (Su et al., 2023; Su et al., 2024). These edited, non‐transgenic citrus plants marked a milestone in agricultural CRISPR applications, receiving United States Department of Agriculture (USDA) Animal and Plant Health Inspection Service (APHIS) and Environmental Protection Agency (EPA) clearance for field release (Su et al., 2023). However, protoplast regeneration remains inefficient in many crop species.

As an alternative, *Agrobacterium*‐mediated transient expression of multiplex CRISPR constructs, combined with both positive and negative selection, has shown promise across diverse species. Base editing or gene targeting introduces gain‐of‐function mutations in *ALS* (acetolactate synthase) to confer resistance to sulfonylurea herbicides like chlorsulfuron (Alquézar et al., 2022; Van den Broeck et al., 2025), while simultaneously targeting gene(s) of interest for mutagenesis (Su et al., 2024; Hoengenaert et al., 2025). When paired with a scorable reporter such as GFP, this system enables negative selection against T‐DNA integration events (Figure 1) (Huang et al., 2023). This design has significantly improved the recovery of transgene‐free, biallelic mutants in tobacco, tomato, potato, and citrus in the T_0_ generation by up to two orders of magnitude compared to standard *Agrobacterium* transient expression without selection (Huang et al., 2023).

In poplar, a cytosine base editor was transiently expressed via *Agrobacterium* along with multiple gRNAs targeting two endogenous *ALS* genes and the lignin biosynthesis gene *CCoAOMT1* (caffeoyl*‐CoA O‐methyltransferase1*). Nearly half of the chlorsulfuron‐resistant shoots were transgene‐free, although editing efficiency at the lignin gene was low (Hoengenaert et al., 2025). Incorporating a counter‐selection marker could streamline identification of transgene‐free events and reduce downstream screening efforts. Another study reported the efficient co‐editing of *ALS* and *CEN1* genes, resulting in transgene‐free, early‐flowering poplar (Table 1) (Wu et al., 2025). Together, these findings demonstrate that transgene‐free genome editing can be achieved in the first generation using *Agrobacterium*‐mediated transient expression, offering a scalable and cost‐effective strategy for perennial crops.

### Crossing with doubled haploid induction

In annual crops, transgene‐free edited plants are often obtained by segregating out transgenes through crossing, as discussed in earlier sections. However, somatic chimerism remains a challenge when dealing with multiplex or heterozygous edits, often necessitating multiple generations of crossing and screening to obtain homozygous mutants (Box 2). Combining multiplex CRISPR editing with doubled haploid induction offers a promising solution to reduce chimerism and accelerate the development of homozygous mutant lines.

Doubled haploid production, either via *in vitro* microspore or anther culture, or through haploid inducer lines, can rapidly fix edited alleles by generating homozygous individuals from haploid cells (Qu et al., 2024). When integrated with multiplex editing, this approach enables immediate recovery of uniform, non‐chimeric, and transgene‐free plants, as demonstrated in wheat, maize, and Arabidopsis (Impens et al., 2023; Kelliher et al., 2019; Wang et al., 2019). Haploid inducer lines carrying CRISPR reagents can directly edit elite cultivars that are otherwise recalcitrant to transformation, including intergeneric crosses between wheat ovules and maize inducer pollen, followed by embryo rescue (Kelliher et al., 2019). Alternatively, multiplex‐edited T_0_ plants, such as those derived from the BREEDIT pipeline (Lorenzo et al., 2023), can be backcrossed to wild type to generate heterozygous, segregating populations. Transgene‐free individuals can then be crossed with haploid inducers to recover homozygous mutants with diverse combinations of target gene edits, thereby accelerating quantitative trait improvement (Impens et al., 2023). However, applying this strategy to perennial crops will require a rapid‐cycle flowering system to overcome long generation times.

## TECHNICAL CONSIDERATIONS AND CHALLENGES

### Multiplex construct design

**Figure 2:**
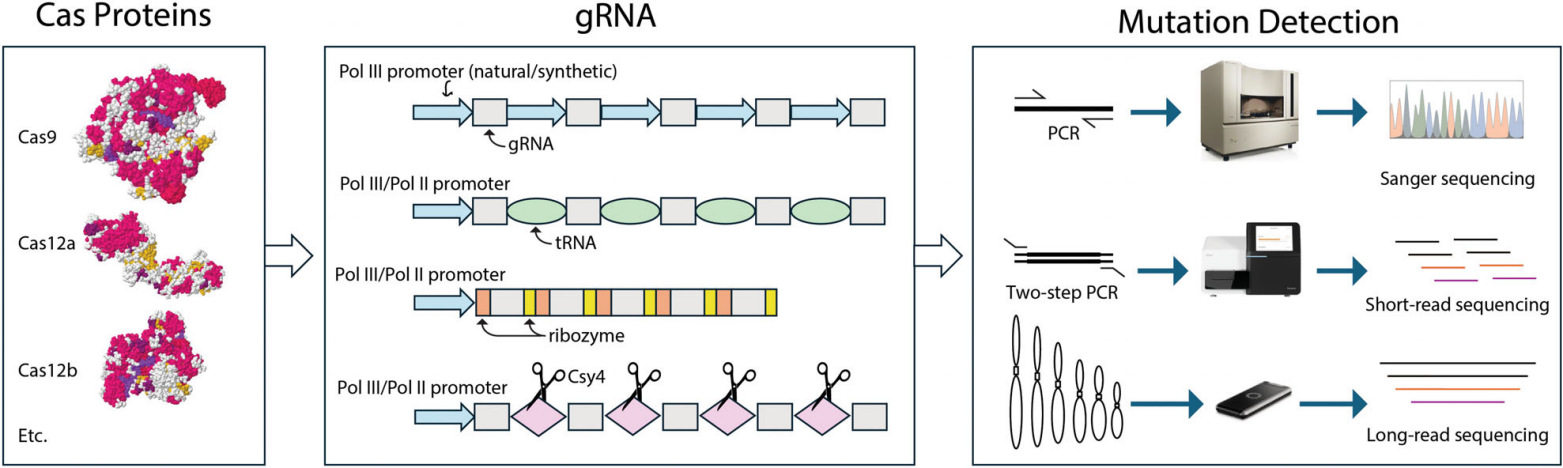
Multiplex CRISPR genome editing reagents and mutation mapping. The left panel depicts commonly used Cas proteins, including Cas9, Cas12a, and Cas12b. The middle panel illustrates various strategies for gRNA expression using different polymerase III (Pol III), Pol II, and hybrid configurations. gRNA can be processed by tRNA‐, ribozyme‐ or Csy4‐based systems. The right panel shows post‐editing mutation detection approaches, including PCR followed by Sanger sequencing, amplicon sequencing via two‐step PCR with short‐read sequencing, and long‐read sequencing for broader genomic analysis.

Our literature mining revealed that the vast majority of multiplex genome editing studies in plants have utilized Cas9 (including nCas9, dCas9, or PAMless variants), with relatively few using Cas12a or Cas12b (Huang et al., 2023; Jordan et al., 2021; Nagy et al., 2025; Pan et al., 2021; Su et al., 2023; Su et al., 2024) (Table 1, Figure 2). This reflects the high efficiency and stability of Cas9 across diverse plant species and applications, from DNA cleavages to transcriptional regulation. However, it also highlights limitations, such as Cas9's large size, which poses challenges for virus‐based vectors with cargo constraints. While the near‐PAMless SpRY variant has expanded the targeting space, slight sequence biases persist (Walton et al., 2020). Exploring the CRISPR‐Cas atlas featuring artificial intelligence (AI)‐generated editors (Ruffolo et al., 2025) can further expand the diversity of Cas variants for plant applications (Box 2). Continued development of multiplex editing toolkits should explore smaller Cas or Cas‐like proteins to broaden delivery options. Incorporating tissue‐specific or inducible promoters (Bollier et al., 2021; Decaestecker et al., 2019; Garcia‐Perez et al., 2022; Gehrke et al., 2023; Zhang et al., 2024) will also enable spatiotemporal control of Cas activity.

As multiplex editing increases in complexity, so does the number of gRNAs that must be expressed. For CRISPR‐Cas9 systems, two common approaches are used: (1) each gRNA is driven by its own promoter, typically a Pol III (Polymerase III) promoter, or (2) a single (Pol II or Pol III) promoter drives expression of a polycistronic gRNA array, with individual gRNAs separated by cleavable RNA elements such as tRNAs (Xie et al., 2015), self‐cleaving ribozymes (Nowak et al., 2016), or recognition sequences for exogenous nucleases like Csy4 (Qi et al., 2012) (Table 1, Figure 2). The first approach allows independent control of each gRNA, and constructs are often built using Gateway or Golden Gate cloning systems and their derivatives (Čermák et al., 2017; Lowder et al., 2015; Ma, Zhang, et al., 2015; Marillonnet & Grützner, 2020; Sarrion‐Perdigones et al., 2011; Zhang et al., 2020). However, repeated use of the same Pol III promoter or inclusion of long promoter sequences can make constructs bulky and difficult to assemble. Recent studies have shown that minimal Pol III promoters—70 bp for dicots (Deguchi et al., 2025) and 160 bp for monocots (Nagy et al., 2025)—are effective for gRNA expression. Using short, non‐redundant promoters not only reduces construct size but also streamlines cloning by enabling low‐cost DNA synthesis for one‐step assembly of multiplex gRNA cassettes directly into binary vectors (Deguchi et al., 2025; Nagy et al., 2025; Ortega et al., 2023).

The second approach uses a single promoter to express multiple gRNAs, simplifying transcriptional control. However, this design inevitably introduces other repetitive elements, such as tRNAs, ribozymes, or Csy4, for gRNA processing, which can offset gains in compactness. The efficiency of these gRNA processing systems can vary by species and construct architecture, requiring empirical optimization (Čermák *et al*., 2017; Cao et al., 2022). Furthermore, not all gRNAs are equally effective (Table 1) (de Vries et al., 2021; Houbaert et al., 2025; Ma, Yang, et al., 2024; Sánchez‐León et al., 2024; Yang et al., 2024). In a few studies, editing or activation efficiencies declined as the number of gRNAs increased (Angulo et al., 2023; Selma et al., 2022; Stuttmann et al., 2021; Xie et al., 2015), though the underlying causes remain unclear. A drop in multiplex editing efficiency was also reported in soybean transformed via a pooled library with a simple and identical construct architecture (one gRNA per construct) (Bai et al., 2020), although potential silencing could not be ruled out in this case due to integration of multiple T‐DNA copies. Unlike Cas9, Cas12a (Cpf1) can self‐process pre‐crRNA arrays, eliminating the need for additional processing elements (Jordan et al., 2021). Nonetheless, independent promoters, tRNA‐gRNA, and ribozyme‐based methods have also been used with Cas12a for multiplex editing (Huang et al., 2023; Nagy et al., 2025).

A less explored yet essential component of CRISPR‐Cas9 multiplex constructs is the gRNA scaffold—a synthetic fusion of crRNA and tracrRNA required for Cas9 binding (Nishimasu et al., 2014). Because each gRNA includes a scaffold, this element becomes highly repetitive in multiplex designs. In *E. coli*, Reis et al. (2019) developed a high‐throughput design‐build‐test‐learn framework that integrated biophysical modeling, experimental characterization, and machine learning (ML) to systematically optimize gRNA scaffold variants using 23 design principles. This approach enabled DNA synthesis and seamless assembly of CRISPR arrays containing up to 22 non‐redundant gRNAs, overcoming scaffold repetitiveness as a key bottleneck in multiplex editing. Similar strategies have been applied in plants, where structure‐guided and random mutagenesis approaches have yielded diverse, functional scaffold variants (Wang, Li, et al., 2024).

The growing adoption of AI and large language models (LLMs) in biological research will further advance CRISPR innovation. For example, the recently developed CRISPR‐GPT enables LLM‐powered automation of the entire CRISPR experimental workflow, from design and planning to execution and data analysis (Qu et al., 2025). Its modular, multi‐agent architecture facilitates human‐AI collaboration, streamlining complex multiplex genome engineering, as demonstrated in proof‐of‐concept gene knockout and epigenetic activation experiments in human cell lines (Qu et al., 2025). Expanding CRISPR‐GPT applications to agricultural crops holds tremendous promise for broadening access to precision genome editing across diverse plant systems (Box 2).

### Mutation determination

A decade ago, when CRISPR was first applied to plants, mutation detection often relied on targeting regions with restriction enzyme sites or designing two gRNAs per gene. This enabled simple PCR‐based genotyping, sometimes followed by restriction enzyme or T7 endonuclease I digestion, before confirming edits via Sanger sequencing (Nekrasov et al., 2013; Shan et al., 2013; Wang, Naik, et al., 2014) (Table 1, Figure 2). Direct sequencing of PCR products was also common, and several computational tools were developed to interpret mixed chromatograms (Brinkman et al., 2014; Conant et al., 2022; Ma, Chen, et al., 2015). While rapid and cost‐effective for small‐scale validation, PCR‐based methods lack the resolution to detect subtle edits or distinguish among multiple gene copies or alleles. Multi‐tier PCR genotyping has been used to identify inversion alleles from TAG editing (Liu et al., 2023), but this modified approach is labor‐ and time‐intensive, requiring context‐dependent primer design that is difficult to scale and ineffective for other structural rearrangements. Sanger sequencing is also inadequate for detecting low‐frequency or complex edits, especially in chimeric tissues, polyploid genomes, or multiplex experiments. Cloning PCR products prior to sequencing can improve allelic resolution but is more resource‐intensive and not scalable.

Amplicon sequencing, typically with the Illumina platform, offers high‐throughput, scalable detection of mutation patterns and editing efficiency at individual target sites (Table 1, Figure 2) (Li et al., 2018; Sánchez‐León et al., 2018; Schmidt et al., 2019; Tang et al., 2017; Wang et al., 2018; Woo et al., 2015; Zhou et al., 2015). For multi‐allelic targets, large deletions that disrupt primer binding sites can be inferred as no‐amplification alleles (Bewg et al., 2022; Chen et al., 2023). When consensus primers are used to amplify highly homologous regions, both on‐ and off‐target effects can be assessed simultaneously (Tsai et al., 2020; Zhou et al., 2015). Several open‐source tools are available to facilitate high‐throughput data processing (Bruyneel et al., 2019; Li et al., 2025; Liu et al., 2019; Park et al., 2016; Xue & Tsai, 2015). A recent review provides additional computational resources, including gRNA design tools, for large‐scale CRISPR experiments (Huang et al., 2022). However, the short‐read length of the Illumina platform (e.g., 2 × 150 bp or 2 × 250 bp) is a limitation, especially when distinguishing between highly homologous paralogs and alleles that lack sufficient sequence variation, such as single‐nucleotide polymorphisms (SNPs). This constraint can limit the available sequence space for gRNA design. Short‐read sequencing is also unable to detect large or complex structural changes, such as inversions and translocations, which are often associated with TAG editing, as discussed earlier.

Target capture sequencing offers a PCR‐free alternative that enables *de novo* assembly and detection of unexpected structural variants, including complex inversions (Table 1) (Chen et al., 2023), within the genomic regions of interest. However, it is more resource‐intensive and may not be practical for routine screening of large populations. Whole‐genome short‐read sequencing has been used to detect chromosomal translocations in Arabidopsis, which has a small genome and high‐quality reference (Table 1) (Beying et al., 2020). This approach is less feasible for heterozygous or polyploid species.

Although not yet widely adopted for CRISPR mutational mapping (Sato et al., 2024), long‐read sequencing technologies—especially the portable and cost‐effective MinION and Flongle devices from Oxford Nanopore Technologies (ONT)—are emerging as powerful tools for structural variation detection in multiplex experiments (Figure 2). When combined with adaptive sampling, these platforms can enable real‐time, targeted enrichment of regions of interest (Martin et al., 2022). With improvements in error rate and sufficient read coverage, ONT sequencing can also detect SNPs and small indels (Sato et al., 2024), making it a promising platform for comprehensive analysis of multiplex editing outcomes.

Whole‐genome sequencing provides the most comprehensive view of both on‐ and off‐target effects. However, it remains cost‐prohibitive for most plant species, particularly those lacking high‐quality reference genomes. The common practice has been to sequence one or a few edited events (Table 1) (Beying et al., 2020; Hendelman et al., 2021; Huang et al., 2023; Jordan et al., 2021; Li et al., 2020; Peterson et al., 2016; Rodríguez‐Leal et al., 2017; Su et al., 2024; Van den Broeck et al., 2025; Wei et al., 2024; Wu et al., 2025; Yu et al., 2024). Even then, off‐target analysis is typically limited to computationally predicted sites, as genome‐wide assessments are confounded by spontaneous mutations and natural polymorphisms. When off‐target potential is minimized during gRNA design, whole‐genome sequencing of edited plants may not be necessary. However, for complex multiplexing or chromosomal engineering, whole‐genome sequencing or optical genome mapping (Table 1) is essential to validate editing outcomes (Schwartz et al., 2020).

Finally, our review of recent publications highlights a shift in the CRISPR field—from system optimization and method validation to its routine use in biological research as a standard and reliable tool. Mutation mapping details and editing metrics are no longer the focal point, but are often abbreviated as part of the routine process of generating a few confirmed mutants to support downstream functional characterization (Table 1) (Shen et al., 2019; Shen et al., 2022; Yu et al., 2021; Zhong et al., 2019). In this evolving context, multiplex editing studies increasingly rely on trait‐based screening rather than exhaustive genotyping, especially when editing outcomes are complex or when phenotypic consequences cannot be reliably inferred from mutation patterns alone. This is particularly central for regulatory sequence editing and multiplex experiments, where combinatorial effects and intermutant interactions may yield unexpected or emergent traits (Lorenzo et al., 2023; Song et al., 2022; Wei et al., 2024; Yang et al., 2024).

## CONCLUSIONS AND FUTURE OUTLOOK

Multiplex gene editing is reshaping the landscape of plant biotechnology and crop improvement. It offers an efficient and scalable alternative to traditional breeding and single‐gene editing by enabling simultaneous and combinatorial manipulation of complex traits. As toolkits become more sophisticated and accessible, and as analytical pipelines improve for detecting complex editing outcomes, multiplex editing is poised to become a core technology in both basic research and applied breeding programs.

Beyond editing genes and regulatory sequences, multiplex approaches will be instrumental in targeting highly repetitive and previously intractable genomic regions, such as transportable elements (Guo et al., 2024), satellite or tandem repeats (Schindele et al., 2022; Tripathi et al., 2019), and heterochromatin (Khosravi et al., 2025; Weiss et al., 2022). These regions hold untapped potential for understanding genome function and unlocking new avenues for trait innovation.

Future efforts should focus on improving gRNA efficiency prediction to enhance editing performance, minimizing off‐target effects, and developing robust and species‐agnostic delivery systems, especially for recalcitrant, perennial, and/or polyploid species. The growing integration of AI, ML, and LLMs into plant breeding and bioengineering research (Farooq et al., 2024; Lam et al., 2024; Yan & Wang, 2022; Zhang et al., 2025) offers powerful tools to streamline experimental design, optimize construct assembly strategy, and predict editing outcomes. These technologies can accelerate every stage of the synthetic biology and plant design workflow, from target gene and gRNA selection to genetic part compatibility, construct building, assay design, and phenotyping, thereby enhancing the speed and precision of design‐build‐test‐learn cycles (Qu et al., 2025). With continued innovation in CRISPR reagents, synthetic biology toolkits, delivery technologies, and computational design pipelines, multiplex editing holds immense promise for harnessing the full potential of plant genome engineering to meet the challenges of sustainable agriculture, food security, energy independence, and climate resilience.

## Conflict of Interest

The authors declare no conflict of interest.

## Data Availability

Data sharing not applicable to this article as no datasets were generated or analysed during the current study.

## References

[tpj70527-bib-0001] Aguirre, L. , Hendelman, A. , Hutton, S.F. , McCandlish, D.M. & Lippman, Z.B. (2023) Idiosyncratic and dose‐dependent epistasis drives variation in tomato fruit size. Science, 382, 315–320. 37856609 10.1126/science.adi5222 PMC10602613

[tpj70527-bib-0002] Alquézar, B. , Bennici, S. , Carmona, L. , Gentile, A. & Peña, L. (2022) Generation of transfer‐DNA‐free base‐edited citrus plants. Frontiers in Plant Science, 13, 835282. 35371165 10.3389/fpls.2022.835282 PMC8965368

[tpj70527-bib-0003] Angulo, J. , Astin, C.P. , Bauer, O. , Blash, K.J. , Bowen, N.M. , Chukwudinma, N.J. et al. (2023) CRISPR/Cas9 mutagenesis of the Arabidopsis GROWTH‐REGULATING FACTOR (GRF) gene family. Frontiers in Genome Editing, 5, 1251557. 37908969 10.3389/fgeed.2023.1251557 PMC10613670

[tpj70527-bib-0004] Assaad, F.F. , Tucker, K.L. & Signer, E.R. (1993) Epigenetic repeat‐induced gene silencing (RIGS) in Arabidopsis. Plant Molecular Biology, 22, 1067–1085. 8400126 10.1007/BF00028978

[tpj70527-bib-0005] Bai, M. , Yuan, J. , Kuang, H. , Gong, P. , Li, S. , Zhang, Z. et al. (2020) Generation of a multiplex mutagenesis population via pooled CRISPR‐Cas9 in soya bean. Plant Biotechnology Journal, 18, 721–731. 31452351 10.1111/pbi.13239 PMC7004907

[tpj70527-bib-0006] Bewg, W.P. , Harding, S.A. , Engle, N.L. , Vaidya, B.N. , Zhou, R. , Reeves, J. et al. (2022) Multiplex knockout of trichome‐regulating MYB duplicates in hybrid poplar using a single gRNA. Plant Physiology, 189, 516–526. 35298644 10.1093/plphys/kiac128 PMC9157173

[tpj70527-bib-0007] Beying, N. , Schmidt, C. , Pacher, M. , Houben, A. & Puchta, H. (2020) CRISPR–Cas9‐mediated induction of heritable chromosomal translocations in *Arabidopsis* . Nature Plants, 6, 638–645. 32451449 10.1038/s41477-020-0663-x

[tpj70527-bib-0008] Biswas, S. , Ibarra, O. , Shaphek, M. , Molina‐Risco, M. , Faion‐Molina, M. , Bellinatti‐Della Gracia, M. et al. (2023) Increasing the level of resistant starch in ‘presidio’ rice through multiplex CRISPR‐Cas9 gene editing of starch branching enzyme genes. Plant Genome, 16, e20225. 35713092 10.1002/tpg2.20225 PMC12806894

[tpj70527-bib-0009] Boerjan, W. & Strauss, S.H. (2024) Social and biological innovations are essential to deliver transformative forest biotechnologies. The New Phytologist, 243, 526–536. 38803120 10.1111/nph.19855

[tpj70527-bib-0010] Bollier, N. , Andrade Buono, R. , Jacobs, T.B. & Nowack, M.K. (2021) Efficient simultaneous mutagenesis of multiple genes in specific plant tissues by multiplex CRISPR. Plant Biotechnology Journal, 19, 651–653. 33305496 10.1111/pbi.13525 PMC8051595

[tpj70527-bib-0011] Brandizzi, F. , Mortimer, J. & Denby, K. (2025) Plant engineering: advances, bottlenecks, and promise. The Plant Journal, 122, e70117. 40275776 10.1111/tpj.70117 PMC12204594

[tpj70527-bib-0012] Brinkman, E.K. , Chen, T. , Amendola, M. & van Steensel, B. (2014) Easy quantitative assessment of genome editing by sequence trace decomposition. Nucleic Acids Research, 42, e168. 25300484 10.1093/nar/gku936 PMC4267669

[tpj70527-bib-0013] Bruyneel, A.A.N. , Colas, A.R. , Karakikes, I. & Mercola, M. (2019) AlleleProfileR: a versatile tool to identify and profile sequence variants in edited genomes. PLoS One, 14, e0226694. 31877162 10.1371/journal.pone.0226694 PMC6932767

[tpj70527-bib-0014] Büschges, R. , Hollricher, K. , Panstruga, R. , Simons, G. , Wolter, M. , Frijters, A. et al. (1997) The barley *Mlo* gene: a novel control element of plant pathogen resistance. Cell, 88, 695–705. 9054509 10.1016/s0092-8674(00)81912-1

[tpj70527-bib-0015] Bzymek, M. & Lovett, S.T. (2001) Instability of repetitive DNA sequences: the role of replication in multiple mechanisms. Proceedings of the National Academy of Sciences, 98, 8319–8325. 10.1073/pnas.111008398 PMC37438 11459970

[tpj70527-bib-0016] Camerlengo, F. , Frittelli, A. , Sparks, C. , Doherty, A. , Martignago, D. , Larré, C. et al. (2020) CRISPR‐Cas9 multiplex editing of the α‐amylase/trypsin inhibitor genes to reduce allergen proteins in durum wheat. Frontiers in Sustainable Food Systems, 4, 104.

[tpj70527-bib-0017] Cao, L. , Wang, Z. , Ma, H. , Liu, T. , Ji, J. & Duan, K. (2022) Multiplex CRISPR/Cas9‐mediated raffinose synthase gene editing reduces raffinose family oligosaccharides in soybean. Frontiers in Plant Science, 13, 1048967. 36457532 10.3389/fpls.2022.1048967 PMC9706108

[tpj70527-bib-0018] Carrère, S. , Routaboul, J.M. , Savourat, P. , Bellenot, C. , López, H. , Sahoo, A. et al. (2024) A fully sequenced collection of homozygous EMS mutants for forward and reverse genetic screens in Arabidopsis thaliana. Plant Journal, 119, 3015–3026. 10.1111/tpj.16954 39073886

[tpj70527-bib-0019] Casas‐Mollano, J.A. , Zinselmeier, M.H. , Sychla, A. & Smanski, M.J. (2023) Efficient gene activation in plants by the MoonTag programmable transcriptional activator. Nucleic Acids Research, 51, 7083–7093. 37254802 10.1093/nar/gkad458 PMC10359618

[tpj70527-bib-0020] Čermák, T. , Curtin, S.J. , Gil‐Humanes, J. , Čegan, R. , Kono, T.J.Y. , Konečná, E. et al. (2017) A multipurpose toolkit to enable advanced genome engineering in plants. Plant Cell, 29, 1196–1217. 28522548 10.1105/tpc.16.00922 PMC5502448

[tpj70527-bib-0021] Chen, Y.‐H. , Sharma, S. , Bewg, W.P. , Xue, L.‐J. , Gizelbach, C.R. & Tsai, C.‐J. (2023) Multiplex editing of the Nucleoredoxin1 tandem array in poplar: from small indels to translocations and complex inversions. The CRISPR Journal, 6, 339–349. 37307061 10.1089/crispr.2022.0096 PMC10460964

[tpj70527-bib-0022] Conant, D. , Hsiau, T. , Rossi, N. , Oki, J. , Maures, T. , Waite, K. et al. (2022) Inference of CRISPR edits from sanger trace data. The CRISPR Journal, 5, 123–130. 35119294 10.1089/crispr.2021.0113

[tpj70527-bib-0023] Cong, L. , Ran, F.A. , Cox, D. , Lin, S.L. , Barretto, R. , Habib, N. et al. (2013) Multiplex genome engineering using CRISPR/Cas systems. Science, 339, 819–823. 23287718 10.1126/science.1231143 PMC3795411

[tpj70527-bib-0024] Consonni, C. , Humphry, M.E. , Hartmann, H.A. , Livaja, M. , Durner, J. , Westphal, L. et al. (2006) Conserved requirement for a plant host cell protein in powdery mildew pathogenesis. Nature Genetics, 38, 716–720. 16732289 10.1038/ng1806

[tpj70527-bib-0025] de Vries, L. , Brouckaert, M. , Chanoca, A. , Kim, H. , Regner, M.R. , Timokhin, V.I. et al. (2021) CRISPR‐Cas9 editing of CAFFEOYL SHIKIMATE ESTERASE 1 and 2 shows their importance and partial redundancy in lignification in *Populus tremula* × *P. alba* . Plant Biotechnology Journal, 19, 2221–2234. 34160888 10.1111/pbi.13651 PMC8541784

[tpj70527-bib-0026] Decaestecker, W. , Buono, R.A. , Pfeiffer, M.L. , Vangheluwe, N. , Jourquin, J. , Karimi, M. et al. (2019) CRISPR‐TSKO: a technique for efficient mutagenesis in specific cell types, tissues, or organs in *Arabidopsis* . The Plant Cell, 31, 2868–2887. 31562216 10.1105/tpc.19.00454 PMC6925012

[tpj70527-bib-0027] Deguchi, M. , Sinclair, K.M. , Patel, A. , Coile, M. , Ortega, M.A. , Bewg, W.P. et al. (2025) A compendium of nonredundant short polymerase III promoters for CRISPR applications. Plant Physiology, 198, kiaf294. 40673482 10.1093/plphys/kiaf294 PMC12268498

[tpj70527-bib-0028] Eid, A. , Mohan, C. , Sanchez, S. , Wang, D. & Altpeter, F. (2021) Multiallelic, targeted mutagenesis of magnesium chelatase with CRISPR/Cas9 provides a rapidly scorable phenotype in highly polyploid sugarcane. Frontiers in Genome Editing, 3, 654996. 34713257 10.3389/fgeed.2021.654996 PMC8525377

[tpj70527-bib-0029] Fagny, M. & Austerlitz, F. (2021) Polygenic adaptation: integrating population genetics and gene regulatory networks. Trends in Genetics, 37, 631–638. 33892958 10.1016/j.tig.2021.03.005

[tpj70527-bib-0030] Farooq, M.A. , Gao, S. , Hassan, M.A. , Huang, Z. , Rasheed, A. , Hearne, S. et al. (2024) Artificial intelligence in plant breeding. Trends in Genetics, 40, 891–908. 39117482 10.1016/j.tig.2024.07.001

[tpj70527-bib-0031] Flagel, L.E. & Wendel, J.F. (2009) Gene duplication and evolutionary novelty in plants. New Phytologist, 183, 557–564. 19555435 10.1111/j.1469-8137.2009.02923.x

[tpj70527-bib-0032] Gallego‐Bartolomé, J. , Gardiner, J. , Liu, W. , Papikian, A. , Ghoshal, B. , Kuo, H.Y. et al. (2018) Targeted DNA demethylation of the *Arabidopsis* genome using the human TET1 catalytic domain. Proceedings of the National Academy of Sciences, 115, E2125–E2134. 10.1073/pnas.1716945115 PMC5834696 29444862

[tpj70527-bib-0033] Garcia‐Perez, E. , Diego‐Martin, B. , Quijano‐Rubio, A. , Moreno‐Giménez, E. , Selma, S. , Orzaez, D. et al. (2022) A copper switch for inducing CRISPR/Cas9‐based transcriptional activation tightly regulates gene expression in Nicotiana benthamiana. BMC Biotechnology, 22, 12. 35331211 10.1186/s12896-022-00741-x PMC8943966

[tpj70527-bib-0034] Gardiner, J. , Ghoshal, B. , Wang, M. & Jacobsen, S.E. (2022) CRISPR–Cas‐mediated transcriptional control and epi‐mutagenesis. Plant Physiology, 188, 1811–1824. 35134247 10.1093/plphys/kiac033 PMC8968285

[tpj70527-bib-0035] Gayral, P. , Noa‐Carrazana, J.C. , Lescot, M. , Lheureux, F. , Lockhart, B.E. , Matsumoto, T. et al. (2008) A single Banana streak virus integration event in the banana genome as the origin of infectious endogenous pararetrovirus. Journal of Virology, 82, 6697–6710. 18417582 10.1128/JVI.00212-08 PMC2447048

[tpj70527-bib-0036] Gehrke, F. , Ruiz‐Duarte, P. , Schindele, A. , Wolf, S. & Puchta, H. (2023) An inducible CRISPR‐kill system for temporally controlled cell type‐specific cell ablation in Arabidopsis thaliana. New Phytologist, 239, 2041–2052. 37381079 10.1111/nph.19102

[tpj70527-bib-0037] Ghoshal, B. , Picard, C.L. , Vong, B. , Feng, S. & Jacobsen, S.E. (2021) CRISPR‐based targeting of DNA methylation in *Arabidopsis thaliana* by a bacterial CG‐specific DNA methyltransferase. Proceedings of the National Academy of Sciences of the United States of America, 118, e2125016118. 34074795 10.1073/pnas.2125016118 PMC8201958

[tpj70527-bib-0038] Gilbertson, L. , Puchta, H. & Slotkin, R.K. (2025) The future of genome editing in plants. Nature Plants, 11, 680–685. 40169873 10.1038/s41477-025-01956-4

[tpj70527-bib-0039] Guo, Y. , Xue, Z. , Gong, M. , Jin, S. , Wu, X. & Liu, W. (2024) CRISPR‐TE: a web‐based tool to generate single guide RNAs targeting transposable elements. Mobile DNA, 15, 3. 38303094 10.1186/s13100-024-00313-0 PMC10832116

[tpj70527-bib-0040] Hanada, K. , Zou, C. , Lehti‐Shiu, M.D. , Shinozaki, K. & Shiu, S.H. (2008) Importance of lineage‐specific expansion of plant tandem duplicates in the adaptive response to environmental stimuli. Plant Physiology, 148, 993–1003. 18715958 10.1104/pp.108.122457 PMC2556807

[tpj70527-bib-0041] Hendelman, A. , Zebell, S. , Rodriguez‐Leal, D. , Dukler, N. , Robitaille, G. , Wu, X. et al. (2021) Conserved pleiotropy of an ancient plant homeobox gene uncovered by *cis*‐regulatory dissection. Cell, 184, 1724–1739.e1716. 33667348 10.1016/j.cell.2021.02.001

[tpj70527-bib-0042] Hoengenaert, L. , Anders, C. , Van Doorsselaere, J. , Vanholme, R. & Boerjan, W. (2025) Transgene‐free genome editing in poplar. New Phytologist, 247, 224–232. 39841625 10.1111/nph.20415

[tpj70527-bib-0043] Hoenicka, H. , Lehnhardt, D. , Briones, V. , Nilsson, O. & Fladung, M. (2016) Low temperatures are required to induce the development of fertile flowers in transgenic male and female early flowering poplar (*Populus tremula* L.). Tree Physiology, 36, 667–677. 27052434 10.1093/treephys/tpw015 PMC4886290

[tpj70527-bib-0044] Houbaert, A. , Denervaud Tendon, V. , Hoermayer, L. , Morffy, N. , Strader, L.C. & Geldner, N. (2025) Efficient, cell‐type‐specific production of flavonols by multiplexed CRISPR activation of a suite of metabolic enzymes. Nature Communications, 16, 6559. 10.1038/s41467-025-61742-w PMC12267567 40670339

[tpj70527-bib-0045] Huang, X. , Jia, H. , Xu, J. , Wang, Y. , Wen, J. & Wang, N. (2023) Transgene‐free genome editing of vegetatively propagated and perennial plant species in the T0 generation via a co‐editing strategy. Nature Plants, 9, 1591–1597. 37723203 10.1038/s41477-023-01520-y

[tpj70527-bib-0046] Huang, Y. , Shang, M. , Liu, T. & Wang, K. (2022) High‐throughput methods for genome editing: the more the better. Plant Physiology, 188, 1731–1745. 35134245 10.1093/plphys/kiac017 PMC8968257

[tpj70527-bib-0047] Impens, L. , Lorenzo, C.D. , Vandeputte, W. , Wytynck, P. , Debray, K. , Haeghebaert, J. et al. (2023) Combining multiplex gene editing and doubled haploid technology in maize. New Phytologist, 239, 1521–1532. 37306056 10.1111/nph.19021 PMC7614789

[tpj70527-bib-0048] Iohannes, S.D. & Jackson, D. (2023) Tackling redundancy: genetic mechanisms underlying paralog compensation in plants. The New Phytologist, 240, 1381–1389. 37724752 10.1111/nph.19267

[tpj70527-bib-0049] Ivanov, M. , Buddle, E.A. & Ankeny, R.A. (2025) Regulation as key to fulfilling the promises of agricultural genomics: going beyond bottlenecks in plant gene technology development. The Plant Journal, 122, e70277. 40522595 10.1111/tpj.70277 PMC12169205

[tpj70527-bib-0050] Jander, G. & Barth, C. (2007) Tandem gene arrays: a challenge for functional genomics. Trends in Plant Science, 12, 203–210. 17416543 10.1016/j.tplants.2007.03.008

[tpj70527-bib-0051] Jansing, J. , Sack, M. , Augustine, S.M. , Fischer, R. & Bortesi, L. (2019) CRISPR/Cas9‐mediated knockout of six glycosyltransferase genes in Nicotiana benthamiana for the production of recombinant proteins lacking β‐1,2‐xylose and core α‐1,3‐fucose. Plant Biotechnology Journal, 17, 350–361. 29969180 10.1111/pbi.12981 PMC6335070

[tpj70527-bib-0052] Jordan, W.T. , Currie, S. & Schmitz, R.J. (2021) Multiplex genome editing in Arabidopsis thaliana using Mb3Cas12a. Plant Direct, 5, e344. 34514290 10.1002/pld3.344 PMC8421513

[tpj70527-bib-0053] Kelliher, T. , Starr, D. , Su, X. , Tang, G. , Chen, Z. , Carter, J. et al. (2019) One‐step genome editing of elite crop germplasm during haploid induction. Nature Biotechnology, 37, 287–292. 10.1038/s41587-019-0038-x 30833776

[tpj70527-bib-0054] Khan, M.A. , Herring, G. , Zhu, J.Y. , Oliva, M. , Fourie, E. , Johnston, B. et al. (2025) CRISPRi‐based circuits to control gene expression in plants. Nature Biotechnology, 43, 416–430. 10.1038/s41587-024-02236-w 38769424

[tpj70527-bib-0055] Khosravi, S. , Hinrichs, R. , Rönspies, M. , Haghi, R. , Puchta, H. & Houben, A. (2025) Epigenetic state and gene expression remain stable after CRISPR/Cas‐mediated chromosomal inversions. New Phytologist, 245, 2527–2539. 39878102 10.1111/nph.20403 PMC11840415

[tpj70527-bib-0056] Kiani, S. , Chavez, A. , Tuttle, M. , Hall, R.N. , Chari, R. , Ter‐Ovanesyan, D. et al. (2015) Cas9 gRNA engineering for genome editing, activation and repression. Nature Methods, 12, 1051–1054. 26344044 10.1038/nmeth.3580 PMC4666719

[tpj70527-bib-0057] Kim, Y. , Schumaker, K.S. & Zhu, J.K. (2006) EMS mutagenesis of Arabidopsis. Methods in Molecular Biology, 323, 101–103. 16739570 10.1385/1-59745-003-0:101

[tpj70527-bib-0058] Konermann, S. , Brigham, M.D. , Trevino, A.E. , Joung, J. , Abudayyeh, O.O. , Barcena, C. et al. (2015) Genome‐scale transcriptional activation by an engineered CRISPR‐Cas9 complex. Nature, 517, 583–588. 25494202 10.1038/nature14136 PMC4420636

[tpj70527-bib-0059] Krysan, P.J. , Young, J.C. & Sussman, M.R. (1999) T‐DNA as an insertional mutagen in *Arabidopsis* . The Plant Cell, 11, 2283–2290. 10590158 10.1105/tpc.11.12.2283 PMC144136

[tpj70527-bib-0060] Kumar, K. , Mandal, S.N. , Pradhan, B. , Kaur, P. , Kaur, K. & Neelam, K. (2022) From evolution to revolution: accelerating crop domestication through genome editing. Plant & Cell Physiology, 63, 1607–1623. 36018059 10.1093/pcp/pcac124

[tpj70527-bib-0061] Lam, H.Y.I. , Ong, X.E. & Mutwil, M. (2024) Large language models in plant biology. Trends in Plant Science, 29, 1145–1155. 38797656 10.1016/j.tplants.2024.04.013

[tpj70527-bib-0062] Lee, J.E. , Neumann, M. , Duro, D.I. & Schmid, M. (2019) CRISPR‐based tools for targeted transcriptional and epigenetic regulation in plants. PLoS One, 14, e0222778. 31557222 10.1371/journal.pone.0222778 PMC6762090

[tpj70527-bib-0063] Lee, K. & Wang, K. (2023) Strategies for genotype‐flexible plant transformation. Current Opinion in Biotechnology, 79, 102848. 36463838 10.1016/j.copbio.2022.102848

[tpj70527-bib-0064] Lemmon, Z.H. , Reem, N.T. , Dalrymple, J. , Soyk, S. , Swartwood, K.E. , Rodriguez‐Leal, D. et al. (2018) Rapid improvement of domestication traits in an orphan crop by genome editing. Nature Plants, 4, 766–770. 30287957 10.1038/s41477-018-0259-x

[tpj70527-bib-0065] Li, A. , Jia, S. , Yobi, A. , Ge, Z. , Sato, S.J. , Zhang, C. et al. (2018) Editing of an alpha‐kafirin gene family increases, digestibility and protein quality in sorghum. Plant Physiology, 177, 1425–1438. 29925584 10.1104/pp.18.00200 PMC6084649

[tpj70527-bib-0066] Li, C. , Zong, Y. , Jin, S. , Zhu, H. , Lin, D. , Li, S. et al. (2020) SWISS: multiplexed orthogonal genome editing in plants with a Cas9 nickase and engineered CRISPR RNA scaffolds. Genome Biology, 21, 141. 32546280 10.1186/s13059-020-02051-x PMC7296638

[tpj70527-bib-0067] Li, F. , Tan, X. , Li, S. , Chen, S. , Liu, L. , Huang, J. et al. (2025) SuperDecode: an integrated toolkit for analyzing mutations induced by genome editing. Molecular Plant, 18, 690–702. 40045573 10.1016/j.molp.2025.03.002

[tpj70527-bib-0068] Li, J. , Jiao, G. , Sun, Y. , Chen, J. , Zhong, Y. , Yan, L. et al. (2021) Modification of starch composition, structure and properties through editing of TaSBEIIa in both winter and spring wheat varieties by CRISPR/Cas9. Plant Biotechnology Journal, 19, 937–951. 33236499 10.1111/pbi.13519 PMC8131058

[tpj70527-bib-0069] Li, S. , Shen, L. , Hu, P. , Liu, Q. , Zhu, X. , Qian, Q. et al. (2019) Developing disease‐resistant thermosensitive male sterile rice by multiplex gene editing. Journal of Integrative Plant Biology, 61, 1201–1205. 30623600 10.1111/jipb.12774

[tpj70527-bib-0070] Liu, J. , Wang, F.‐Z. , Li, C. , Li, Y. & Li, J.‐F. (2023) Hidden prevalence of deletion‐inversion bi‐alleles in CRISPR‐mediated deletions of tandemly arrayed genes in plants. Nature Communications, 14, 6787. 10.1038/s41467-023-42490-1 PMC10600118 37880225

[tpj70527-bib-0071] Liu, L. , Gallagher, J. , Arevalo, E.D. , Chen, R. , Skopelitis, T. , Wu, Q. et al. (2021) Enhancing grain‐yield‐related traits by CRISPR‐Cas9 promoter editing of maize CLE genes. Nature Plants, 7, 287–294. 33619356 10.1038/s41477-021-00858-5

[tpj70527-bib-0072] Liu, Q. , Wang, C. , Jiao, X. , Zhang, H. , Song, L. , Li, Y. et al. (2019) Hi‐TOM: a platform for high‐throughput tracking of mutations induced by CRISPR/Cas systems. Science China Life Sciences, 62, 1–7. 30446870 10.1007/s11427-018-9402-9

[tpj70527-bib-0073] Lorenzo, C.D. , Debray, K. , Herwegh, D. , Develtere, W. , Impens, L. , Schaumont, D. et al. (2023) BREEDIT: a multiplex genome editing strategy to improve complex quantitative traits in maize. The Plant Cell, 35, 218–238. 36066192 10.1093/plcell/koac243 PMC9806654

[tpj70527-bib-0074] Lowder, L.G. , Zhang, D. , Baltes, N.J. , Paul, J.W., III , Tang, X. , Zheng, X. et al. (2015) A CRISPR/Cas9 toolbox for multiplexed plant genome editing and transcriptional regulation. Plant Physiology, 169, 971–985. 26297141 10.1104/pp.15.00636 PMC4587453

[tpj70527-bib-0075] Luo, J. , Li, S. , Xu, J. , Yan, L. , Ma, Y. & Xia, L. (2021) Pyramiding favorable alleles in an elite wheat variety in one generation by CRISPR‐Cas9‐mediated multiplex gene editing. Molecular Plant, 14, 847–850. 33812982 10.1016/j.molp.2021.03.024

[tpj70527-bib-0076] Luo, J. , Liu, H. , Zhou, T. , Gu, B. , Huang, X. , Shangguan, Y. et al. (2013) An‐1 encodes a basic helix‐loop‐helix protein that regulates awn development, grain size, and grain number in rice. Plant Cell, 25, 3360–3376. 24076974 10.1105/tpc.113.113589 PMC3809537

[tpj70527-bib-0077] Ma, M. , Yang, L. , Hu, Z. , Mo, C. , Geng, S. , Zhao, X. et al. (2024) Multiplex gene editing reveals cucumber MILDEW RESISTANCE LOCUS O family roles in powdery mildew resistance. Plant Physiology, 195, 1069–1088. 38330431 10.1093/plphys/kiae047

[tpj70527-bib-0078] Ma, P.‐F. , Liu, Y.‐L. , Guo, C. , Jin, G. , Guo, Z.‐H. , Mao, L. et al. (2024) Genome assemblies of 11 bamboo species highlight diversification induced by dynamic subgenome dominance. Nature Genetics, 56, 710–720. 38491323 10.1038/s41588-024-01683-0 PMC11018529

[tpj70527-bib-0079] Ma, X. , Chen, L. , Zhu, Q. , Chen, Y. & Liu, Y.‐G. (2015) Rapid decoding of sequence‐specific nuclease‐induced heterozygous and biallelic mutations by direct sequencing of PCR products. Molecular Plant, 8, 1285–1287. 25747846 10.1016/j.molp.2015.02.012

[tpj70527-bib-0080] Ma, X. , Zhang, Q. , Zhu, Q. , Liu, W. , Chen, Y. , Qiu, R. et al. (2015) A robust CRISPR/Cas9 system for convenient, high‐efficiency multiplex genome editing in monocot and dicot plants. Molecular Plant, 8, 1274–1284. 25917172 10.1016/j.molp.2015.04.007

[tpj70527-bib-0081] Marillonnet, S. & Grützner, R. (2020) Synthetic DNA assembly using golden gate cloning and the hierarchical modular cloning pipeline. Current Protocols in Molecular Biology, 130, e115. 32159931 10.1002/cpmb.115

[tpj70527-bib-0082] Martin, S. , Heavens, D. , Lan, Y. , Horsfield, S. , Clark, M.D. & Leggett, R.M. (2022) Nanopore adaptive sampling: a tool for enrichment of low abundance species in metagenomic samples. Genome Biology, 23, 11. 35067223 10.1186/s13059-021-02582-x PMC8785595

[tpj70527-bib-0083] McCarty, N.S. , Graham, A.E. , Studená, L. & Ledesma‐Amaro, R. (2020) Multiplexed CRISPR technologies for gene editing and transcriptional regulation. Nature Communications, 11, 1281. 10.1038/s41467-020-15053-x PMC7062760 32152313

[tpj70527-bib-0084] Mojica, F.J.M. , Díez‐Villaseñor, C.s. , García‐Martínez, J. & Soria, E. (2005) Intervening sequences of regularly spaced prokaryotic repeats derive from foreign genetic elements. Journal of Molecular Evolution, 60, 174–182. 15791728 10.1007/s00239-004-0046-3

[tpj70527-bib-0085] Müller, N.A. , Kersten, B. , Leite Montalvão, A.P. , Mähler, N. , Bernhardsson, C. , Bräutigam, K. et al. (2020) A single gene underlies the dynamic evolution of poplar sex determination. Nature Plants, 6, 630–637. 32483326 10.1038/s41477-020-0672-9

[tpj70527-bib-0086] Nagy, E.D. , Davis, I.W. , Song, S. , No, V. , Wu, C. , Kanizay, L. et al. (2025) Computationally derived RNA polymerase III promoters enable maize genome editing. Frontiers in Plant Science, 16, 1540425. 40177017 10.3389/fpls.2025.1540425 PMC11961915

[tpj70527-bib-0087] Nasti, R.A. & Voytas, D.F. (2021) Attaining the promise of plant gene editing at scale. Proceedings of the National Academy of Sciences of the United States of America, 118, e2004846117. 34050019 10.1073/pnas.2004846117 PMC8179185

[tpj70527-bib-0088] Nekrasov, V. , Staskawicz, B. , Weigel, D. , Jones, J.D.G. & Kamoun, S. (2013) Targeted mutagenesis in the model plant Nicotiana benthamiana using Cas9 RNA‐guided endonuclease. Nature Biotechnology, 31, 691–693. 10.1038/nbt.2655 23929340

[tpj70527-bib-0089] Nishimasu, H. , Ran, F.A. , Hsu, P.D. , Konermann, S. , Shehata, S.I. , Dohmae, N. et al. (2014) Crystal structure of Cas9 in complex with guide RNA and target DNA. Cell, 156, 935–949. 24529477 10.1016/j.cell.2014.02.001 PMC4139937

[tpj70527-bib-0090] Nowak, C.M. , Lawson, S. , Zerez, M. & Bleris, L. (2016) Guide RNA engineering for versatile Cas9 functionality. Nucleic Acids Research, 44, 9555–9564. 27733506 10.1093/nar/gkw908 PMC5175371

[tpj70527-bib-0091] Ortega, M.A. , Zhou, R. , Chen, M.S.S. , Bewg, W.P. , Simon, B. & Tsai, C.‐J. (2023) In vitro floral development in poplar: insights into seed trichome regulation and trimonoecy. New Phytologist, 237, 1078–1081. 36385612 10.1111/nph.18624 PMC10107547

[tpj70527-bib-0092] Otto, M. , Zheng, Y. & Wiehe, T. (2022) Recombination, selection, and the evolution of tandem gene arrays. Genetics, 221, iyac052. 35460227 10.1093/genetics/iyac052 PMC9252282

[tpj70527-bib-0093] Pan, C. , Li, G. , Malzahn, A.A. , Cheng, Y. , Leyson, B. , Sretenovic, S. et al. (2022) Boosting plant genome editing with a versatile CRISPR‐combo system. Nature Plants, 8, 513–525. 35596077 10.1038/s41477-022-01151-9

[tpj70527-bib-0094] Pan, C. , Wu, X. , Markel, K. , Malzahn, A.A. , Kundagrami, N. , Sretenovic, S. et al. (2021) CRISPR–Act3.0 for highly efficient multiplexed gene activation in plants. Nature Plants, 7, 942–953. 34168320 10.1038/s41477-021-00953-7

[tpj70527-bib-0095] Park, J. , Lim, K. , Kim, J.‐S. & Bae, S. (2016) Cas‐analyzer: an online tool for assessing genome editing results using NGS data. Bioinformatics, 33, 286–288. 27559154 10.1093/bioinformatics/btw561 PMC5254075

[tpj70527-bib-0096] Peterson, B.A. , Haak, D.C. , Nishimura, M.T. , Teixeira, P.J.P.L. , James, S.R. , Dangl, J.L. et al. (2016) Genome‐wide assessment of efficiency and specificity in CRISPR/Cas9 mediated multiple site targeting in Arabidopsis. PLoS One, 11, e0162169. 27622539 10.1371/journal.pone.0162169 PMC5021288

[tpj70527-bib-0097] Piatek, A. , Ali, Z. , Baazim, H. , Li, L. , Abulfaraj, A. , Al‐Shareef, S. et al. (2015) RNA‐guided transcriptional regulation in planta via synthetic dCas9‐based transcription factors. Plant Biotechnology Journal, 13, 578–589. 25400128 10.1111/pbi.12284

[tpj70527-bib-0098] Qi, L. , Haurwitz, R.E. , Shao, W. , Doudna, J.A. & Arkin, A.P. (2012) RNA processing enables predictable programming of gene expression. Nature Biotechnology, 30, 1002–1006. 10.1038/nbt.2355 22983090

[tpj70527-bib-0099] Qu, Y. , Fernie, A.R. , Liu, J. & Yan, J. (2024) Doubled haploid technology and synthetic apomixis: recent advances and applications in future crop breeding. Molecular Plant, 17, 1005–1018. 38877700 10.1016/j.molp.2024.06.005

[tpj70527-bib-0100] Qu, Y. , Huang, K. , Yin, M. , Zhan, K. , Liu, D. , Yin, D. et al. (2025) CRISPR‐GPT for agentic automation of gene‐editing experiments. *Nature* . Biomedical Engineering. 10.1038/s41551-025-01463-z PMC12920143 40738974

[tpj70527-bib-0101] Quiroz, L.F. , Khan, M. , Gondalia, N. , Lai, L. , McKeown, P.C. , Brychkova, G. et al. (2024) Tissue culture‐independent approaches to revolutionizing plant transformation and gene editing. Horticulture Research, 12, uhae292. 39906168 10.1093/hr/uhae292 PMC11789523

[tpj70527-bib-0102] Raabe, K. , Sun, L. , Schindfessel, C. , Honys, D. & Geelen, D. (2024) A word of caution: T‐DNA‐associated mutagenesis in plant reproduction research. Journal of Experimental Botany, 75, 3248–3258. 38477707 10.1093/jxb/erae114

[tpj70527-bib-0103] Reis, A.C. , Halper, S.M. , Vezeau, G.E. , Cetnar, D.P. , Hossain, A. , Clauer, P.R. et al. (2019) Simultaneous repression of multiple bacterial genes using nonrepetitive extra‐long sgRNA arrays. Nature Biotechnology, 37, 1294–1301. 10.1038/s41587-019-0286-9 31591552

[tpj70527-bib-0104] Rizzon, C. , Ponger, L. & Gaut, B.S. (2006) Striking similarities in the genomic distribution of tandemly arrayed genes in *Arabidopsis* and rice. PLoS Computational Biology, 2, e115. 16948529 10.1371/journal.pcbi.0020115 PMC1557586

[tpj70527-bib-0105] Roca Paixão, J.F. , Gillet, F.‐X. , Ribeiro, T.P. , Bournaud, C. , Lourenço‐Tessutti, I.T. , Noriega, D.D. et al. (2019) Improved drought stress tolerance in *Arabidopsis* by CRISPR/dCas9 fusion with a histone acetyltransferase. Scientific Reports, 9, 8080. 31147630 10.1038/s41598-019-44571-y PMC6542788

[tpj70527-bib-0106] Rodríguez‐Leal, D. , Lemmon, Z.H. , Man, J. , Bartlett, M.E. & Lippman, Z.B. (2017) Engineering quantitative trait variation for crop improvement by genome editing. Cell, 171, 470–480.e478. 28919077 10.1016/j.cell.2017.08.030

[tpj70527-bib-0107] Rodriguez‐Leal, D. , Xu, C. , Kwon, C.‐T. , Soyars, C. , Demesa‐Arevalo, E. , Man, J. et al. (2019) Evolution of buffering in a genetic circuit controlling plant stem cell proliferation. Nature Genetics, 51, 786–792. 30988512 10.1038/s41588-019-0389-8 PMC7274162

[tpj70527-bib-0108] Rönspies, M. , Schmidt, C. , Schindele, P. , Lieberman‐Lazarovich, M. , Houben, A. & Puchta, H. (2022) Massive crossover suppression by CRISPR‐Cas‐mediated plant chromosome engineering. Nature Plants, 8, 1153–1159. 36109610 10.1038/s41477-022-01238-3

[tpj70527-bib-0109] Ruffolo, J.A. , Nayfach, S. , Gallagher, J. , Bhatnagar, A. , Beazer, J. , Hussain, R. et al. (2025) Design of highly functional genome editors by modelling CRISPR–Cas sequences. Nature, 645, 518–525. 40739342 10.1038/s41586-025-09298-z PMC12422970

[tpj70527-bib-0110] Samach, A. , Mafessoni, F. , Gross, O. , Melamed‐Bessudo, C. , Filler‐Hayut, S. , Dahan‐Meir, T. et al. (2023) CRISPR/Cas9‐induced DNA breaks trigger crossover, chromosomal loss, and chromothripsis‐like rearrangements. The Plant Cell, 35, 3957–3972. 37497643 10.1093/plcell/koad209 PMC10615209

[tpj70527-bib-0111] Sánchez‐León, S. , Gil‐Humanes, J. , Ozuna, C.V. , Giménez, M.J. , Sousa, C. , Voytas, D.F. et al. (2018) Low‐gluten, nontransgenic wheat engineered with CRISPR/Cas9. Plant Biotechnology Journal, 16, 902–910. 28921815 10.1111/pbi.12837 PMC5867031

[tpj70527-bib-0112] Sánchez‐León, S. , Marín‐Sanz, M. , Guzmán‐López, M.H. , Gavilán‐Camacho, M. , Simón, E. & Barro, F. (2024) CRISPR/Cas9‐mediated multiplex gene editing of gamma and omega gliadins: paving the way for gliadin‐free wheat. Journal of Experimental Botany, 75, 7079–7095. 39238167 10.1093/jxb/erae376 PMC11630021

[tpj70527-bib-0113] Sarrion‐Perdigones, A. , Falconi, E.E. , Zandalinas, S.I. , Juárez, P. , Fernández‐del‐Carmen, A. , Granell, A. et al. (2011) GoldenBraid: an iterative cloning system for standardized assembly of reusable genetic modules. PLoS One, 6, e21622. 21750718 10.1371/journal.pone.0021622 PMC3131274

[tpj70527-bib-0114] Sato, R. , Nanasato, Y. , Takata, N. , Nagano, S. , Fukatsu, E. , Fujino, T. et al. (2024) Efficient selection of a biallelic and nonchimeric gene‐edited tree using Oxford nanopore technologies sequencing. Tree Physiology, 44, tpad158. 38145493 10.1093/treephys/tpad158

[tpj70527-bib-0115] Schindele, A. , Gehrke, F. , Schmidt, C. , Röhrig, S. , Dorn, A. & Puchta, H. (2022) Using CRISPR‐kill for organ specific cell elimination by cleavage of tandem repeats. Nature Communications, 13, 1502. 10.1038/s41467-022-29130-w PMC8938420 35314679

[tpj70527-bib-0116] Schmidt, C. , Fransz, P. , Rönspies, M. , Dreissig, S. , Fuchs, J. , Heckmann, S. et al. (2020) Changing local recombination patterns in Arabidopsis by CRISPR/Cas mediated chromosome engineering. Nature Communications, 11, 4418. 10.1038/s41467-020-18277-z PMC7474074 32887885

[tpj70527-bib-0117] Schmidt, C. , Pacher, M. & Puchta, H. (2019) Efficient induction of heritable inversions in plant genomes using the CRISPR/Cas system. The Plant Journal, 98, 577–589. 30900787 10.1111/tpj.14322

[tpj70527-bib-0118] Schwartz, C. , Lenderts, B. , Feigenbutz, L. , Barone, P. , Llaca, V. , Fengler, K. et al. (2020) CRISPR–Cas9‐mediated 75.5‐mb inversion in maize. Nature Plants, 6, 1427–1431. 33299151 10.1038/s41477-020-00817-6

[tpj70527-bib-0119] Selma, S. , Bernabé‐Orts, J.M. , Vazquez‐Vilar, M. , Diego‐Martin, B. , Ajenjo, M. , Garcia‐Carpintero, V. et al. (2019) Strong gene activation in plants with genome‐wide specificity using a new orthogonal CRISPR/Cas9‐based programmable transcriptional activator. Plant Biotechnology Journal, 17, 1703–1705. 31034138 10.1111/pbi.13138 PMC6686126

[tpj70527-bib-0120] Selma, S. , Sanmartín, N. , Espinosa‐Ruiz, A. , Gianoglio, S. , Lopez‐Gresa, M.P. , Vázquez‐Vilar, M. et al. (2022) Custom‐made design of metabolite composition in *N. benthamiana* leaves using CRISPR activators. Plant Biotechnology Journal, 20, 1578–1590. 35514036 10.1111/pbi.13834 PMC9342607

[tpj70527-bib-0121] Shan, Q. , Wang, Y. , Li, J. , Zhang, Y. , Chen, K. , Liang, Z. et al. (2013) Targeted genome modification of crop plants using a CRISPR‐Cas system. Nature Biotechnology, 31, 686–688. 10.1038/nbt.2650 23929338

[tpj70527-bib-0122] Shelake, R.M. , Kadam, U.S. , Kumar, R. , Pramanik, D. , Singh, A.K. & Kim, J.‐Y. (2022) Engineering drought and salinity tolerance traits in crops through CRISPR‐mediated genome editing: targets, tools, challenges, and perspectives. Plant Communications, 3, 100417. 35927945 10.1016/j.xplc.2022.100417 PMC9700172

[tpj70527-bib-0123] Shen, W. , Liu, J. & Li, J.‐F. (2019) Type‐II metacaspases mediate the processing of plant elicitor peptides in *Arabidopsis* . Molecular Plant, 12, 1524–1533. 31454707 10.1016/j.molp.2019.08.003

[tpj70527-bib-0124] Shen, W. , Zhang, X. , Liu, J. , Tao, K. , Li, C. , Xiao, S. et al. (2022) Plant elicitor peptide signalling confers rice resistance to piercing‐sucking insect herbivores and pathogens. Plant Biotechnology Journal, 20, 991–1005. 35068048 10.1111/pbi.13781 PMC9055822

[tpj70527-bib-0125] Song, X. , Meng, X. , Guo, H. , Cheng, Q. , Jing, Y. , Chen, M. et al. (2022) Targeting a gene regulatory element enhances rice grain yield by decoupling panicle number and size. Nature Biotechnology, 40, 1403–1411. 10.1038/s41587-022-01281-7 35449414

[tpj70527-bib-0126] Souza, G.M. , Van Sluys, M.‐A. , Lembke, C.G. , Lee, H. , Margarido, G.R.A. , Hotta, C.T. et al. (2019) Assembly of the 373k gene space of the polyploid sugarcane genome reveals reservoirs of functional diversity in the world's leading biomass crop. GigaScience, 8, giz129. 31782791 10.1093/gigascience/giz129 PMC6884061

[tpj70527-bib-0127] Staginnus, C. & Richert‐Pöggeler, K.R. (2006) Endogenous pararetroviruses: two‐faced travelers in the plant genome. Trends in Plant Science, 11, 485–491. 16949329 10.1016/j.tplants.2006.08.008

[tpj70527-bib-0128] Stuttmann, J. , Barthel, K. , Martin, P. , Ordon, J. , Erickson, J.L. , Herr, R. et al. (2021) Highly efficient multiplex editing: one‐shot generation of 8× Nicotiana benthamiana and 12× Arabidopsis mutants. The Plant Journal, 106, 8–22. 33577114 10.1111/tpj.15197

[tpj70527-bib-0129] Su, H. , Wang, Y. , Xu, J. , Omar, A.A. , Grosser, J.W. , Calovic, M. et al. (2023) Generation of the transgene‐free canker‐resistant *Citrus sinensis* using Cas12a/crRNA ribonucleoprotein in the T0 generation. Nature Communications, 14, 3957. 10.1038/s41467-023-39714-9 PMC10319737 37402755

[tpj70527-bib-0130] Su, H. , Wang, Y. , Xu, J. , Omar, A.A. , Grosser, J.W. & Wang, N. (2024) Cas12a RNP‐mediated co‐transformation enables transgene‐free multiplex genome editing, long deletions, and inversions in citrus chromosome. Frontiers in Plant Science, 15, 1448807. 39148610 10.3389/fpls.2024.1448807 PMC11324552

[tpj70527-bib-0131] Sulis, D.B. , Jiang, X. , Yang, C. , Marques, B.M. , Matthews, M.L. , Miller, Z. et al. (2023) Multiplex CRISPR editing of wood for sustainable fiber production. Science, 381, 216–221. 37440632 10.1126/science.add4514 PMC10542590

[tpj70527-bib-0132] Tanenbaum, M.E. , Gilbert, L.A. , Qi, L.S. , Weissman, J.S. & Vale, R.D. (2014) A protein‐tagging system for signal amplification in gene expression and fluorescence imaging. Cell, 159, 635–646. 25307933 10.1016/j.cell.2014.09.039 PMC4252608

[tpj70527-bib-0133] Tang, X. , Lowder, L.G. , Zhang, T. , Malzahn, A.A. , Zheng, X. , Voytas, D.F. et al. (2017) A CRISPR‐Cpf1 system for efficient genome editing and transcriptional repression in plants. Nature Plants, 3, 17018. 28211909 10.1038/nplants.2017.18

[tpj70527-bib-0134] Tripathi, J.N. , Ntui, V.O. , Ron, M. , Muiruri, S.K. , Britt, A. & Tripathi, L. (2019) CRISPR/Cas9 editing of endogenous banana streak virus in the B genome of Musa spp. overcomes a major challenge in banana breeding. Communications Biology, 2, 46. 30729184 10.1038/s42003-019-0288-7 PMC6355771

[tpj70527-bib-0135] Tsai, C.‐J. , Xu, P. , Xue, L.‐J. , Hu, H. , Nyamdari, B. , Naran, R. et al. (2020) Compensatory guaiacyl lignin biosynthesis at the expense of syringyl lignin in *4CL1*‐knockout poplar. Plant Physiology, 183, 123–136. 32139476 10.1104/pp.19.01550 PMC7210618

[tpj70527-bib-0136] Van den Broeck, S. , Ngapout, Y. , Panis, B. & Vanderschuren, H. (2025) An agrobacterium‐mediated base editing approach generates transgene‐free edited banana. New Phytologist, 247, 1790–1804. 40170227 10.1111/nph.70044

[tpj70527-bib-0137] Walton, R.T. , Christie, K.A. , Whittaker, M.N. & Kleinstiver, B.P. (2020) Unconstrained genome targeting with near‐PAMless engineered CRISPR‐Cas9 variants. Science, 368, 290–296. 32217751 10.1126/science.aba8853 PMC7297043

[tpj70527-bib-0138] Wang, B. , Liu, X. , Li, Z. , Zeng, K. , Guo, J. , Xin, T. et al. (2024) A nuclease‐dead Cas9‐derived tool represses target gene expression. Plant Physiology, 195, 1880–1892. 38478589 10.1093/plphys/kiae149

[tpj70527-bib-0139] Wang, B. , Zhu, L. , Zhao, B. , Zhao, Y. , Xie, Y. , Zheng, Z. et al. (2019) Development of a haploid‐inducer mediated genome editing system for accelerating maize breeding. Molecular Plant, 12, 597–602. 30902686 10.1016/j.molp.2019.03.006

[tpj70527-bib-0140] Wang, J.P. , Naik, P.P. , Chen, H.‐C. , Shi, R. , Lin, C.‐Y. , Liu, J. et al. (2014) Complete proteomic‐based enzyme reaction and inhibition kinetics reveal how monolignol biosynthetic enzyme families affect metabolic flux and lignin in *Populus trichocarpa* . Plant Cell, 26, 894–914. 24619611 10.1105/tpc.113.120881 PMC4001400

[tpj70527-bib-0141] Wang, J.Y. & Doudna, J.A. (2023) CRISPR technology: a decade of genome editing is only the beginning. Science, 379, eadd8643. 36656942 10.1126/science.add8643

[tpj70527-bib-0142] Wang, W. , Pan, Q. , He, F. , Akhunova, A. , Chao, S. , Trick, H. et al. (2018) Transgenerational CRISPR‐Cas9 activity facilitates multiplex gene editing in allopolyploid wheat. The CRISPR Journal, 1, 65–74. 30627700 10.1089/crispr.2017.0010 PMC6319321

[tpj70527-bib-0143] Wang, X. , Aguirre, L. , Rodríguez‐Leal, D. , Hendelman, A. , Benoit, M. & Lippman, Z.B. (2021) Dissecting cis‐regulatory control of quantitative trait variation in a plant stem cell circuit. Nature Plants, 7, 419–427. 33846596 10.1038/s41477-021-00898-x

[tpj70527-bib-0144] Wang, Y. , Cheng, X. , Shan, Q. , Zhang, Y. , Liu, J. , Gao, C. et al. (2014) Simultaneous editing of three homoeoalleles in hexaploid bread wheat confers heritable resistance to powdery mildew. Nature Biotechnology, 32, 947–951. 10.1038/nbt.2969 25038773

[tpj70527-bib-0145] Wang, Y. , Li, X. , Liu, M. , Zhou, Y. & Li, F. (2024) Guide RNA scaffold variants enabled easy cloning of large gRNA cluster for multiplexed gene editing. Plant Biotechnology Journal, 22, 460–471. 37816147 10.1111/pbi.14198 PMC10826992

[tpj70527-bib-0146] Wei, Y. , Zhang, H. , Fan, J. , Cai, Q. , Zhang, Z. , Wang, J. et al. (2024) Multiplex‐genome‐editing based rapid directional improvement of complex traits in rice. Plant Biotechnology Journal, 22, 2624–2628. 38803114 10.1111/pbi.14375 PMC11331775

[tpj70527-bib-0147] Weiss, T. , Crisp, P.A. , Rai, K.M. , Song, M. , Springer, N.M. & Zhang, F. (2022) Epigenetic features drastically impact CRISPR–Cas9 efficacy in plants. Plant Physiology, 190, 1153–1164. 35689624 10.1093/plphys/kiac285 PMC9516779

[tpj70527-bib-0148] Wolter, F. , Schindele, P. & Puchta, H. (2019) Plant breeding at the speed of light: the power of CRISPR/Cas to generate directed genetic diversity at multiple sites. BMC Plant Biology, 19, 176. 31046670 10.1186/s12870-019-1775-1 PMC6498546

[tpj70527-bib-0149] Woo, J.W. , Kim, J. , Kwon, S.I. , Corvalan, C. , Cho, S.W. , Kim, H. et al. (2015) DNA‐free genome editing in plants with preassembled CRISPR‐Cas9 ribonucleoproteins. Nature Biotechnology, 33, 1162–1164. 10.1038/nbt.3389 26479191

[tpj70527-bib-0150] Wu, L. , Zhong, L. , Xiao, H. , Cao, F. & Cheng, Q. (2025) Generation of transgene‐free homozygous early‐flowering poplar via cytosine base editing. Plant Physiology, 198, kiaf290. 40660944 10.1093/plphys/kiaf290

[tpj70527-bib-0151] Xie, K. , Minkenberg, B. & Yang, Y. (2015) Boosting CRISPR/Cas9 multiplex editing capability with the endogenous tRNA‐processing system. Proceedings of the National Academy of Sciences, 112, 3570–3575. 10.1073/pnas.1420294112 PMC4371917 25733849

[tpj70527-bib-0152] Xu, L. , Sun, B. , Liu, S. , Gao, X. , Zhou, H. , Li, F. et al. (2023) The evaluation of active transcriptional repressor domain for CRISPRi in plants. Gene, 851, 146967. 36261092 10.1016/j.gene.2022.146967

[tpj70527-bib-0153] Xue, L.‐J. & Tsai, C.‐J. (2015) AGEseq: analysis of genome editing by sequencing. Molecular Plant, 8, 1428–1430. 26057235 10.1016/j.molp.2015.06.001

[tpj70527-bib-0154] Yan, J. & Wang, X. (2022) Unsupervised and semi‐supervised learning: the next frontier in machine learning for plant systems biology. The Plant Journal, 111, 1527–1538. 35821601 10.1111/tpj.15905

[tpj70527-bib-0155] Yang, L. , Güell, M. , Niu, D. , George, H. , Lesha, E. , Grishin, D. et al. (2015) Genome‐wide inactivation of porcine endogenous retroviruses (PERVs). Science, 350, 1101–1104. 26456528 10.1126/science.aad1191

[tpj70527-bib-0156] Yang, Q. , Ral, J.‐P. , Wei, Y. , Zheng, Y. , Li, Z. & Jiang, Q. (2024) Genome editing of five starch synthesis genes produces highly resistant starch and dietary fibre in barley grains. Plant Biotechnology Journal, 22, 2051–2053. 38415905 10.1111/pbi.14324 PMC11182577

[tpj70527-bib-0157] Yang, T. , Ali, M. , Lin, L. , Li, P. , He, H. , Zhu, Q. et al. (2023) Recoloring tomato fruit by CRISPR/Cas9‐mediated multiplex gene editing. Horticulture Research, 10, uhac214. 36643741 10.1093/hr/uhac214 PMC9832834

[tpj70527-bib-0158] Ye, S. , Chen, G. , Kohnen, M.V. , Wang, W. , Cai, C. , Ding, W. et al. (2020) Robust CRISPR/Cas9 mediated genome editing and its application in manipulating plant height in the first generation of hexaploid Ma bamboo (Dendrocalamus latiflorus Munro). Plant Biotechnology Journal, 18, 1501–1503. 31858701 10.1111/pbi.13320 PMC7292545

[tpj70527-bib-0159] Yu, H. , Lin, T. , Meng, X. , Du, H. , Zhang, J. , Liu, G. et al. (2021) A route to *de novo* domestication of wild allotetraploid rice. Cell, 184, 1156–1170.e1114. 33539781 10.1016/j.cell.2021.01.013

[tpj70527-bib-0160] Yu, J. , Ke, T. , Tehrim, S. , Sun, F. , Liao, B. & Hua, W. (2015) PTGBase: an integrated database to study tandem duplicated genes in plants. Database: The Journal of Biological Databases and Curation, 2015, bav017. 25797062 10.1093/database/bav017 PMC4369376

[tpj70527-bib-0161] Yu, Z. , Yunusbaev, U. , Fritz, A. , Tilley, M. , Akhunova, A. , Trick, H. et al. (2024) CRISPR‐based editing of the ω‐ and γ‐gliadin gene clusters reduces wheat immunoreactivity without affecting grain protein quality. Plant Biotechnology Journal, 22, 892–903. 37975410 10.1111/pbi.14231 PMC10955484

[tpj70527-bib-0162] Zeng, D. , Liu, T. , Ma, X. , Wang, B. , Zheng, Z. , Zhang, Y. et al. (2020) Quantitative regulation of waxy expression by CRISPR/Cas9‐based promoter and 5'UTR‐intron editing improves grain quality in rice. Plant Biotechnology Journal, 18, 2385–2387. 32485068 10.1111/pbi.13427 PMC7680535

[tpj70527-bib-0163] Zhang, C. , Tang, Y. , Tang, S. , Chen, L. , Li, T. , Yuan, H. et al. (2024) An inducible CRISPR activation tool for accelerating plant regeneration. Plant Communications, 5, 100823. 38243597 10.1016/j.xplc.2024.100823 PMC11121170

[tpj70527-bib-0164] Zhang, D. , Xu, F. , Wang, F. , Le, L. & Pu, L. (2025) Synthetic biology and artificial intelligence in crop improvement. Plant Communications, 6, 101220. 39668563 10.1016/j.xplc.2024.101220 PMC11897457

[tpj70527-bib-0165] Zhang, Y. , Held, M.A. , Kaur, D. & Showalter, A.M. (2021) CRISPR‐Cas9 multiplex genome editing of the hydroxyproline‐O‐galactosyltransferase gene family alters arabinogalactan‐protein glycosylation and function in Arabidopsis. BMC Plant Biology, 21, 16. 33407116 10.1186/s12870-020-02791-9 PMC7789275

[tpj70527-bib-0166] Zhang, Y. , Held, M.A. & Showalter, A.M. (2020) Elucidating the roles of three β‐glucuronosyltransferases (GLCATs) acting on arabinogalactan‐proteins using a CRISPR‐Cas9 multiplexing approach in Arabidopsis. BMC Plant Biology, 20, 221. 32423474 10.1186/s12870-020-02420-5 PMC7236193

[tpj70527-bib-0167] Zhong, S. , Liu, M. , Wang, Z. , Huang, Q. , Hou, S. , Xu, Y.‐C. et al. (2019) Cysteine‐rich peptides promote interspecific genetic isolation in *Arabidopsis* . Science, 364, eaau9564. 31147494 10.1126/science.aau9564 PMC7184628

[tpj70527-bib-0168] Zhou, R. , Jenkins, J.W. , Zeng, Y. , Shu, S. , Jang, H. , Harding, S.A. et al. (2023) Haplotype‐resolved genome assembly of *Populus tremula* × *P. alba* reveals aspen‐specific megabase satellite DNA. Plant Journal, 16, 1003–1017. 10.1111/tpj.16454 37675609

[tpj70527-bib-0169] Zhou, X. , Jacobs, T.B. , Xue, L.‐J. , Harding, S.A. & Tsai, C.‐J. (2015) Exploiting SNPs for biallelic CRISPR mutations in the outcrossing woody perennial *populus* reveals 4‐coumarate:CoA ligase specificity and redundancy. New Phytologist, 208, 298–301. 25970829 10.1111/nph.13470

[tpj70527-bib-0170] Zinselmeier, M.H. , Casas‐Mollano, J.A. , Cors, J. , Sychla, A. , Heinsch, S.C. , Voytas, D.F. et al. (2024) Optimized dCas9 programmable transcriptional activators for plants. Plant Biotechnology Journal, 22, 3202–3204. 39058765 10.1111/pbi.14441 PMC11495988

[tpj70527-bib-0171] Zsögön, A. , Čermák, T. , Naves, E.R. , Notini, M.M. , Edel, K.H. , Weinl, S. et al. (2018) De novo domestication of wild tomato using genome editing. Nature Biotechnology, 36, 1211–1216. 10.1038/nbt.4272 30272678

